# Eosinophils and Bioactive Lipid Mediators Regulate Skin Inflammation and Cancer Growth

**DOI:** 10.1016/j.jid.2025.04.015

**Published:** 2025-05-02

**Authors:** Christina Malactou, Mark Hayes, Tingfeng Zhang, Sophie Ward, Catherine Harwood, Jesmond Dalli, Jessica Strid

**Affiliations:** 1 Department of Immunology and Inflammation, Imperial College London, London, United Kingdom; 2 Centre for Cell Biology and Cutaneous Research, Queen Mary University of London, London, United Kingdom; 3 William Harvey Research Institute, Barts and The London School of Medicine and Dentistry, Queen Mary University of London, London, United Kingdom; 4 Centre for Inflammation and Therapeutic Innovation, Queen Mary University of London, London, United Kingdom

**Keywords:** Bioactive lipid mediators, Cancer, Eosinophils, Inflammation, Skin

## Abstract

Cutaneous squamous cell carcinoma (cSCC) is the second most common form of skin cancer, with local immunity playing a key role in regulating outcome. Nonresolving inflammation has been suggested to drive cancer growth; however, a fundamental understanding of the tumor immune microenvironment in cSCC is still lacking. In this study, we demonstrate tissue-associated tumor eosinophilia in both human and murine cSCC and show that eosinophils promote epithelial hyperplasia and tumor outgrowth. The skin eosinophils display tissue adaptation and dynamically alter their transcriptome according to the microenvironment. Furthermore, we show that the skin bioactive lipidome is significantly altered during inflammation and in cSCC and that this is regulated by eosinophils. Inflamed skin and cSCC tissue are dominated by lipid mediators synthesized by lipoxygenase enzymes, and our results suggest that eosinophils influence the balance between proinflammatory and proresolving lipid mediators. We demonstrate that lipid mediators can regulate epithelial cell expansion, with PCTR1 reducing inflammation-driven growth of tumor cells and eosinophil-driven eoxin C4 blocking this effect. Together, these findings highlight that eosinophils and specific bioactive lipid mediators play important roles in skin biology and carcinogenesis and point to potential previously unreported strategies for treatment of inflammatory disorders and epithelial malignancies.

## Introduction

Skin cancers are the most prevalent form of cancer and include cutaneous squamous cell carcinoma (cSCC), which is the fastest-rising cancer worldwide (Eggermont and Eggermont, 2024; Urban et al, 2021). Although most cSCCs are treatable, a substantial number recur or metastasize (~5%), and these account for approximately 25% of skin cancererelated deaths (Karia et al, 2013). There are few effective treatments for advanced cSCC, with a 5-year survival <30% (Harwood et al, 2016). Remarkably, the frequency of cancer-driver mutations in healthy skin is comparable with that in cSCCs (Martincorena et al, 2015), suggesting that there are prevailing physiological mechanisms that constrain cancer outgrowth.

These mechanisms are poorly characterized, but a key role for local immunity is evident from the dramatic increase in the rate and severity of cSCC among immunosuppressed individuals (Harwood et al, 2013). Treatments for advanced disease are associated with only moderate response rates and poor overall survival (Nagarajan et al, 2019). Although recent clinical trials of antiePD-1 checkpoint inhibitor immunotherapy in cSCC hold promise for the future (Migden et al, 2018), this approach is currently not suitable for many high-risk patients such as organ-transplant recipients, and treatment of advanced cSCC remains an area of important unmet clinical need.

Development of cSCC is underpinned by accumulation of somatic mutations in the originating epithelial cells (ECs), but the fate of mutated cells is strongly influenced by the surrounding microenvironment. Skin has an active immunesurveillance system, and we have previously demonstrated that tissue-resident immune cells provide strong host-protective responses against skin carcinogenesis (Crawford et al, 2018; Dalessandri et al, 2016; Strid et al, 2011, 2008). This potent tumor immune-surveillance system is driven primarily by ECs (Dalessandri and Strid, 2014), which upon tissue damage produce an array of immune-modulating cytokines and bioactive lipid mediators that can initiate controlled inflammation, followed by efficient resolution. Nonresolving immune responses may have detrimental effects, and it is widely accepted that many neoplastic conditions are driven, at least in part, by chronic and often subclinical inflammation (Coussens et al, 2013; Diakos et al, 2014; Mantovani et al, 2008; Marx, 2004). Although the role of cytokines in regulating skin immunity have been studied extensively, little is known of how bioactive lipid mediators contribute to skin homeostasis, inflammation, and cancer.

The involvement of bioactive lipid mediators in noncancerous skin inflammation is well-documented. It has been suggested that in inflammatory skin conditions, such as psoriasis and atopic dermatitis, pathology may be driven by an imbalance between inflammatory and anti-inflammatory bioactive lipid mediators (Kendall and Nicolaou, 2013). These observations raise the possibility that some bioactive lipid metabolites may play a key role in the promotion phase of carcinogenesis, whereas proresolving lipid mediators may aid resolution and tissue health.

Epidermal hyperplasia and skin inflammation are hallmarks of a wide range of skin conditions. Frequently, inflamed skin will be infiltrated by a less-studied population of leukocytes: the eosinophils. The ‘eosinophilic dermatoses’ reveal the scope and powerful manner that eosinophils can affect the skin tissue (Long et al, 2016). Nevertheless, a fundamental understanding of how eosinophils operate in the skin during health and disease is currently lacking. Eosinophils are evolutionary conserved, pleiotropic cells with potent immune-modulatory capacity. They accumulate at sites of tissue damage and can exert potent inflammatory effects through the release of preformed proteins and lipid mediators. Eosinophils are also frequently observed infiltrating many solid tumors, and both pro and antitumor functions have been suggested (Grisaru-Tal et al, 2020; Reichman et al, 2016). Nevertheless, few studies have mechanistically elucidated their function in the tumor microenvironment (TME) and in cancer outcomes.

In this study, we report that eosinophils infiltrate the skin in a CCR3-dependent manner and accumulate in inflamed skin and in cSCC tissue in both human and mouse. Using a chemically induced inflammation-driven model of cSCC as well as transplantable tumor cell lines, we demonstrate that eosinophils promote epithelial hyperplasia and the growth of tumors. We show that eosinophils dynamically alter their transcriptome according to the tissue microenvironment and, furthermore, that the bioactive lipid mediator profile of the skin is significantly altered during inflammation and in cancer and that this is regulated by the presence of eosinophils. Although the cyclooxygenase (COX)-derived lipid mediators are not significantly changed, some lipoxygenase (LOX)-derived mediators are strongly upregulated during inflammation and/or in cSCC tissue. The expressions of protectins, maresins, and eoxins are completely dependent on the presence of eosinophils, and we provide evidence for the biological actions of some of these lipid mediators in directly affecting epithelial turnover and tumor growth. Thus, our study demonstrates that skin eosinophils promote skin inflammation and cancer growth, and we suggest this, in part, is by regulating the balance between key pro and antiinflammatory lipid mediators that directly affects EC turnover.

## Results

### Eosinophils infiltrate inflamed skin in a CCR3-dependent manner

**Figure 1:**
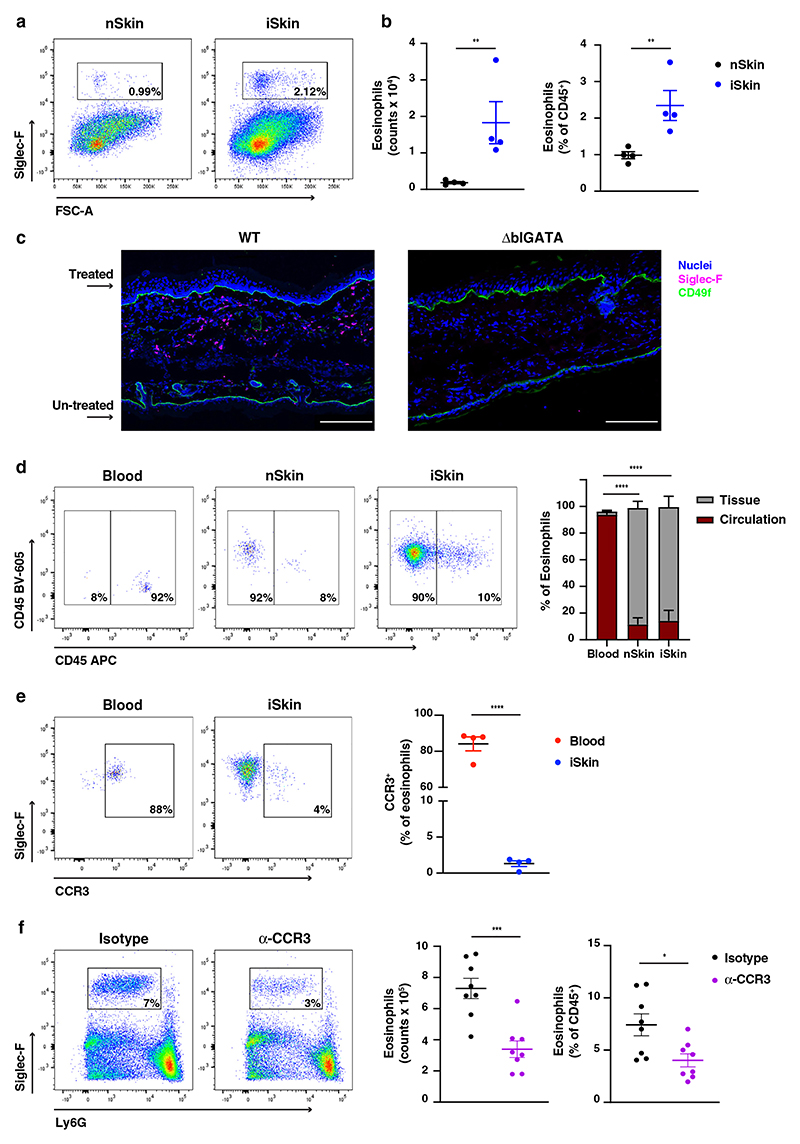
Eosinophils infiltrate inflamed skin in a CCR3-dependent manner. (**a−e**) Mice were exposed topically to the inflammatory agent TPA on the dorsal side of the ear skin (2 times per week for 2 weeks) or left untreated. Single-cell suspensions of whole dorsal skin were analyzed by flow cytometry with eosinophils gated as CD45^+^Siglec-F^+^CD11b^+^ live leukocytes. (**a**) Representative dot plots showing eosinophils gated in nSkin and iSkin. **(b)** Total eosinophil counts in the skin as well as percentage eosinophils of the total CD45^+^ population (n = 4). (**c**) Representative confocal images showing cross-sections of inflamed ear skin of WT and ΔdblGATA mice stained for Siglec-F (pink), CD49f (green), and nuclei visualized with DAPI (blue). A total of 8-μm sections were imaged using a Leica DM4B microscope (× 20). Bar = 100 μm. (**d**) Twenty-four hours after the last TPA treatment, mice were injected i.v. with 5 μg APC-labeled anti-CD45 antibody and culled after 1 min. Ear skin and blood were sampled and stained in vitro for flow cytometry analysis (n = 4). Representative dot plots showing CD45-BV-605e and CD45-APCelabeled eosinophils (gated as SiglecF^+^CD11b^+^) in blood, nSkin, and iSkin, with bar graphs representing percentage of tissue eosinophils (gray bar, CD45-APC^-^ CD45-BV-605^+^) and percentage of i.v.-labeled/circulating eosinophils (red bar, CD45-APC^+^). (**e**) Blood and iSkin eosinophils were analyzed for expression of CCR3 by flow cytometry. (**f**) Mice were injected with 3 μg anti-CCR3 antibody or isotype control i.v. followed by 1 × topical TPA on the dorsal ear skin and a second anti-CCR3 intravenous injection 6 h later. Blood and skin were collected 18 h later and analyzed by flow cytometry; data are shown as representative dot plots, total eosinophil counts, and percentage of eosinophils among total CD45^+^ leukocytes. All data are expressed as mean ± SEM. Statistics was tested by unpaired Student’s *t*-test (for **b, e**, and **f**) and 1-way ANOVA in **d**. *P < .05, **P < .01, ***P < .001, and ****P < .0001. APC, allophycocyanin; h, hour; iSkin, inflamed skin; i.v., intravenously; min, minute; nSkin, naive skin; TPA, 12-O-tetradecanoylphorbol-13-acetate; WT, wild-type.

Eosinophils are a prominent resident immune population in some tissues. Most studies relate to the gastrointestinal tract, so it is of interest to understand their regulation at other bodysurface organs. In the skin, we found that a notable but small population of eosinophils resided in healthy naive skin (nSkin) tissue; however, this population substantially increased in inflamed skin (iSkin) (Figure 1a and b). After topical exposure to agents that induce skin inflammation, such as 12-O-tetradecanoylphorbol-13-acetate (TPA) (a protein kinase C activator), cross-sections of the skin tissue revealed accumulation of eosinophils in the treated inflamed skin but not in the untreated skin and also not in treated skin from ΔdblGATA mice (Figure 1c), which has a targeted mutation in the *Gata1* gene, resulting in a complete lack of eosinophils.

To investigate whether the skin eosinophils were truly tissue resident or were in the enlarged blood vessels in the inflamed skin, we performed in vivo labeling of bloodborne cells, followed by ex vivo labeling of tissue cells. As expected, nearly all eosinophils in the blood were labeled by the in vivo antibody injection, whereas only 8−10% of eosinophils in either nSkin or iSkin were labeled in vivo, indicating that the vast majority was truly tissue resident (Figure 1d). Furthermore, we found that eosinophils in the blood were highly positive for the chemokine receptor CCR3, whereas eosinophils from iSkin were not (Figure 1e). Indeed, blocking CCR3 by systemics administration of antibody significantly reduced eosinophil accumulation in iSkin (Figure 1f), suggesting that blood eosinophils use this receptor to gain access to the iSkin and that they downregulate CCR3 after arrival. Thus, nSkin contains a small population of tissue-resident eosinophils, but their numbers are greatly enhanced during skin inflammation in a CCR3-dependent manner.

### Eosinophils accumulate in cSCC tissue and promote inflammation-driven carcinogenesis

**Figure 2:**
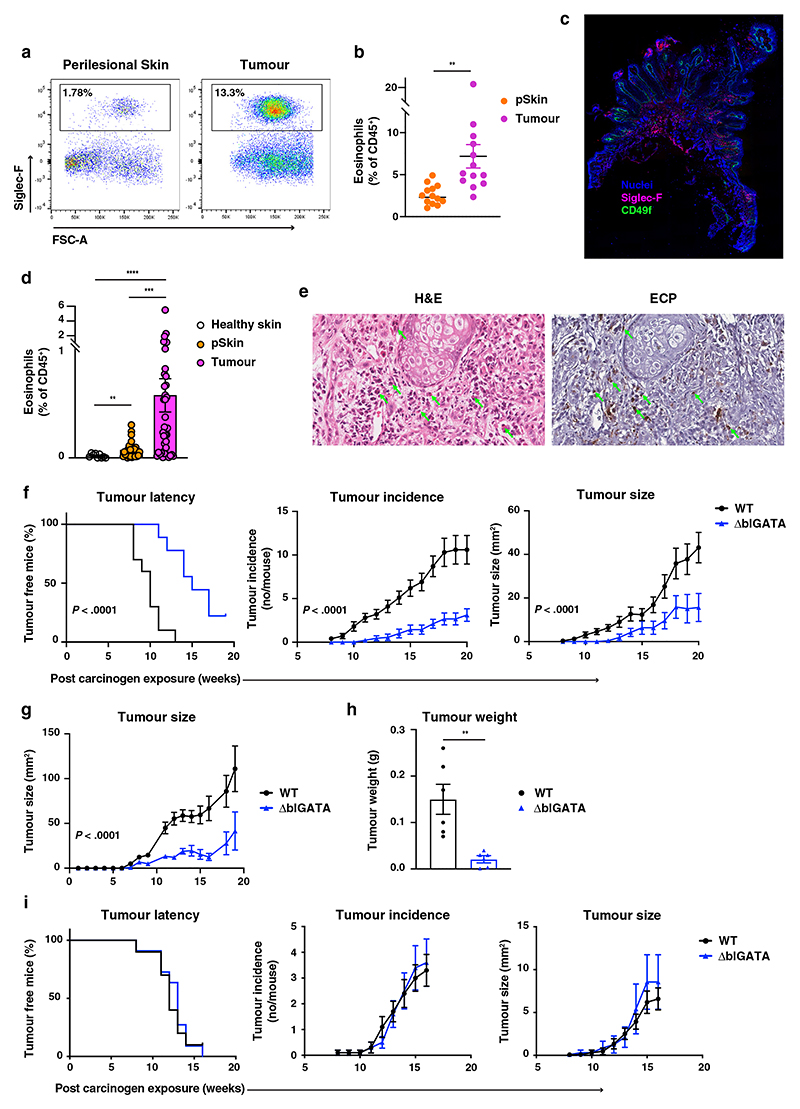
Eosinophils accumulate in cSCC tissue and promote inflammation-driven carcinogenesis. (**a, b**) pSkin and cSCC tumor tissue were collected 20 weeks after DMBA-TPA inflammation-driven carcinogenesis, and single-cell suspensions were analyzed by flow cytometry. (**a**) Representative dot plots with eosinophils gated as CD45^+^Siglec-F^+^CD11b^+^ live leukocytes and (**b**) proportion of eosinophils as percentage of total CD45^+^ populations (n = 13). (**c**) Representative confocal image of a DMBA-TPAeinduced tumor at week 20, with eosinophils visualized by Siglec-F (pink), basement membrane visualized by CD49f (green), and cell nuclei visualized by DAPI (blue). (**d**) Tumor tissues (n = 40) and pSkin (n = 29) surrounding the tumor were collected from human patients with cSCC and healthy excess skin from elective plastic surgery patients (n = 9). Tissue was processed and assessed by flow cytometry for the presence of eosinophils (gated as CD45^+^CD14^-^CD117^-^Siglec8^+^CD11b^+^). (**e)** Representative cross-sections of human cSCC showing immunohistochemical staining by H&E and ECP. (f) WT and eosinophil-deficient (ΔdblGATA) mice underwent DMBA-TPA inflammation-driven carcinogenesis, and tumor susceptibility is expressed as tumor latency (time to appearance of first tumor), tumor incidence (average number of tumors per mouse), and tumor area (average tumor size per mouse) (n = 10 per group). (**g, h**) A total of 10^5^ CT26 cancer cells were transplanted subcutaneously into the right flank of WT and ΔdblGATA mice, and (g) tumor size was monitored over time using callipers, and (**h**) tumors were weighed at the end of experiment (n = 6). (i) WT and ΔdblGATA mice underwent DMBA-only carcinogenesis, and tumor susceptibility was assessed as in **f** (n = 10 per group). Data are expressed as means ± SEM, and statistics were tested by unpaired Student’s *t*-test (for **b** and **h**), 1-way ANOVA (for d), and log-rank (Mantel-Cox) test for tumor latency and linear regression for tumor incidence and area (for **f, g**, and **i**). **P < .01, ***P < .001, and ****P < .0001. cSCC, cutaneous squamous cell carcinoma; DMBA, 7,12-dimethylbenz[a]anthracene; ECP, eosinophil cationic protein; FSC-A, forward scatter area; pSkin, perilesional skin; TPA, 12-O-tetradecanoylphorbol-13-acetate; WT, wild-type.

Persistent and nonresolving inflammation has been suggested to drive cancer growth or progression, but it is less clear which elements of inflammation are involved. Blood eosinophilia was reported in patients with cancer as early as the late 1900s, and tissue-associated tumor eosinophilia has been observed in a variety of solid tumors since then, particularly in mucosal sites such as the gastrointestinal tract and lungs (Grisaru-Tal et al, 2020). In this study, we show that tissue-associated tumor eosinophilia is also a feature of cSCC. First, we used a well-established model of inflammationdriven carcinogenesis (7,12-dimethylbenz[a]anthracene [DMBA]-TPA), in which topical exposure to a subclinical dose of the carcinogen DMBA provokes oncogenic mutations in ECs (not enough for cancer growth) that can subsequently be promoted to grow into overt tumors by chronic tissue inflammation. This model was chosen because it recapitulates features of epithelial carcinogenesis in humans and enables the study of both the initiation and the promotion phase of carcinogenesis (Abel et al, 2009). Analysis of the resulting tumors showed a 10-fold increase in tissue eosinophils within the tumor (Figure 2a and b). The perilesional skin adjacent to the tumor, which had similarly been exposed to the inflammatory agent TPA, also showed an increase in eosinophils compared with the nSkin, albeit this was less marked than in tumor tissue (Figure 2a and b). Cross-sections of the tumors showed that the eosinophils were predominantly in the peritumoral infiltrate, but some had also crossed the basement membrane into the epithelium (Figure 2c). Human cSCC tissue also contained an increased population of eosinophils in both perilesional skin and tumor tissue compared with skin from healthy donors (Figure 2d). Tissue-associated tumor eosinophilia in patients with cSCC could also be visualized by immunohistochemistry, where areas surrounding the tumor cells were rich in eosinophils, seen with their characteristic bilobed nuclei and affinity to the acidophilic dye eosin as well as with granules containing eosinophil cationic protein (Figure 2e). No eosinophilia was detected in the blood of patients with cSCC, with the mean (±SEM) eosinophils of total blood leukocytes at 2.5 ± 0.1% in patients with cSCC and 2.4 ± 0.04% in healthy controls.

To explore whether the eosinophils contributed to carcinogenesis and/or cSCC growth, we compared the susceptibility of wild-type (WT) and eosinophil-deficient (ΔdblGATA) mice in different cancer models. In the 2-stage inflammation-driven model of cSCC (DMBA-TPA), we found that mice with no eosinophils were significantly less susceptible to tumor growth, with increased latency to tumor development and reduced incidence of tumors, and when tumors did develop, they were also smaller (Figure 2f). Despite this significantly altered tumor susceptibility in eosinophil-deficient mice, there were no significant differences in the overall leukocyte infiltrate into the tumors or in the relative abundance of myeloid and lymphoid subpopulations (Supplementary Figure S1a and b). Mice devoid of eosinophils were also less susceptible to tumor development after transplantation of the CT26 cancer cell line, growing significantly smaller tumors as measured both by size over time and by final tumor weight (Figure 2g and h). However, in a model of mutationdriven cutaneous carcinogenesis, without exposure to the inflammatory agent (DMBA only), the absence of eosinophils had no impact on either tumor latency, incidence, or size of the resulting cSCCs (Figure 2i). Together, these data show that eosinophils accumulate in cSCC tissue and promote inflammation-driven carcinogenesis, but they have no impact on cSCC development that is driven by mutational load alone.

### Eosinophils promote hyperplasia of the basal epithelial layer during skin inflammation

**Figure 3:**
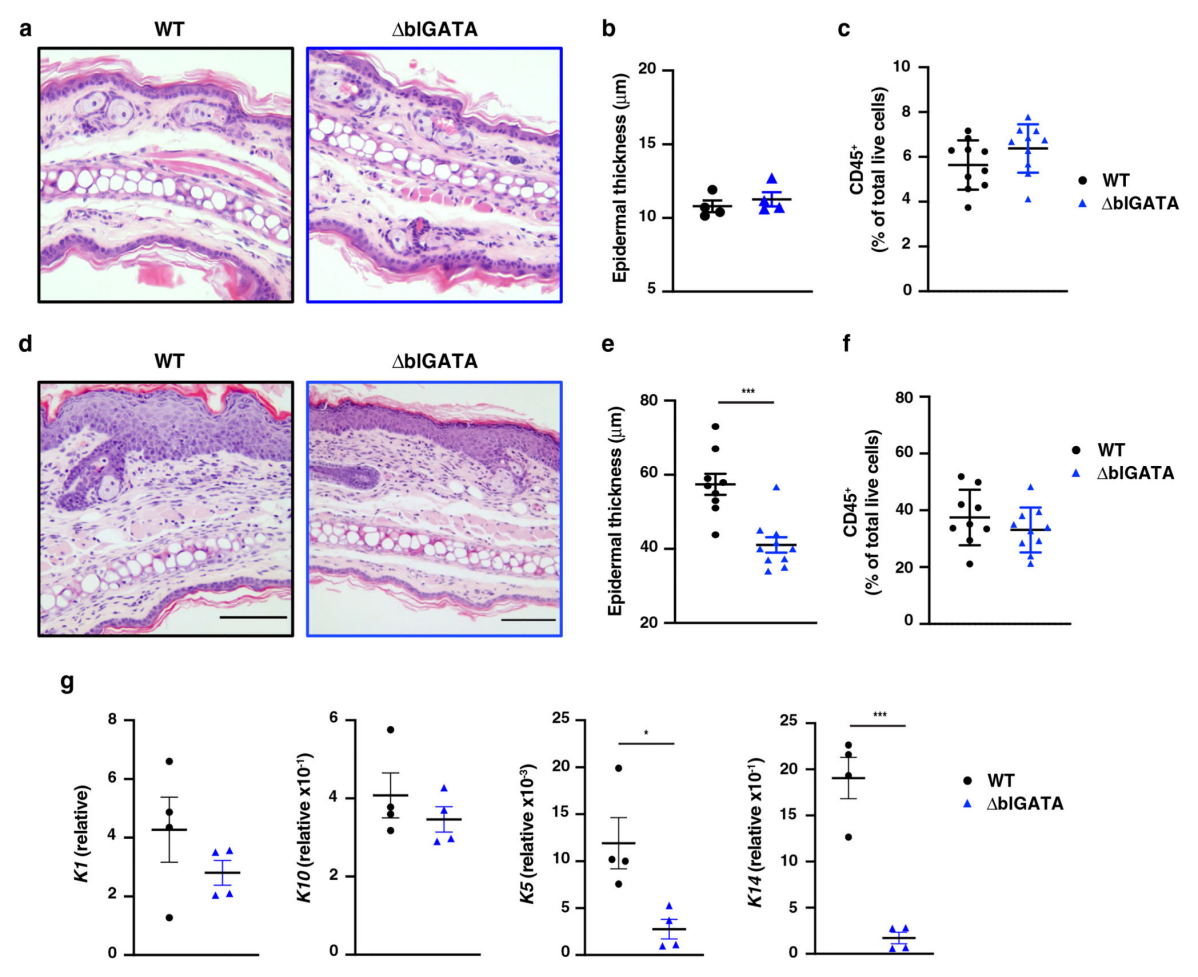
Eosinophils promote hyperplasia of the basal epithelial layer during skin inflammation. WT and ΔdblGATA mice were either left (**a**–**c**) untreated or (**d**−**f**) exposed topically to TPA on the dorsal side of the ear skin (2 times per week for 2 weeks). Forty-eight hours after the last TPA treatment, iSkin and nSkin were collected for immunohistochemistry, flow cytometry, and PCR analysis. Shown are representative images of H&E-stained cross-sections of (**a**) nSkin and (**d**) iSkin. Bar =100 μm. The epidermal thickness of (**b**) nSkin (n = 4) and (**e**) iSkin (WT, n = 9; ΔdblGATA, n = 10) was measured using Fiji software. Total number of skin leukocytes was assessed in (**c**) nSkin (n = 10) and (**f**) iSkin (WT, n = 9; ΔdblGATA n = 10) as percentage CD45^+^ cells among total live cells. (**g**) Expression of mRNA for suprabasal epithelial K1 and K10 and basal epithelial K5 and K14 in the epidermis was analyzed by RT-qPCR and expressed relative to the control gene cyclophilin (n = 4). Data are represented as mean ± SEM, and statistics were tested is by unpaired Student’s *t*-test. *P < .05 and ***P < .001. iSkin, inflamed skin; K1, keratin 1; K10, keratin 10; K14, keratin 14; K5, keratin 5; nSkin, naive skin; TPA, 12-O-tetradecanoylphorbol-13-acetate; WT, wild-type.

There were no gross morphological changes in steady state skin of eosinophil-deficient ΔdblGATA mice or in the total abundance of skin leukocytes (Figure 3a−c). Considering the eosinophil-supported promotion of inflammation-driven carcinogenesis, we next investigated the inflammatory process in isolation. Cross-sections of WT skin after topical exposure to the inflammatory agent TPA showed the classical hallmarks of inflammation with a large immune infiltrate into the dermis and a marked hyperplasia of the epithelium on the exposed skin side. The inflamed skin of mice devoid of eosinophils had an altered morphology with a significantly reduced epidermal hyperplasia (Figure 3d and e) but with an equivalent abundance of infiltrating leukocytes (Figure 3f). There were also no major differences in the relative abundance of myeloid and lymphoid subpopulations—apart from a slight decrease of infiltrating basophils and a concomitant increase in mast cells—in the inflamed skin of eosinophil-deficient mice (Supplementary Figure S2a and b). Furthermore, analysis of the most abundant keratins in the epidermis revealed that the expression of the basal keratin 5 and keratin 14 was significantly reduced during inflammation in eosinophil-deficient mice, whereas there was no difference in the expression of the suprabasal keratin 1 and keratin 10 (Figure 3g). Overall, these findings suggest that eosinophils promote inflammation-induced epidermal hyperplasia by enhancing the level of proliferating basal ECs.

### Eosinophils dynamically alter their phenotype according to the skin microenvironment

**Figure 4:**
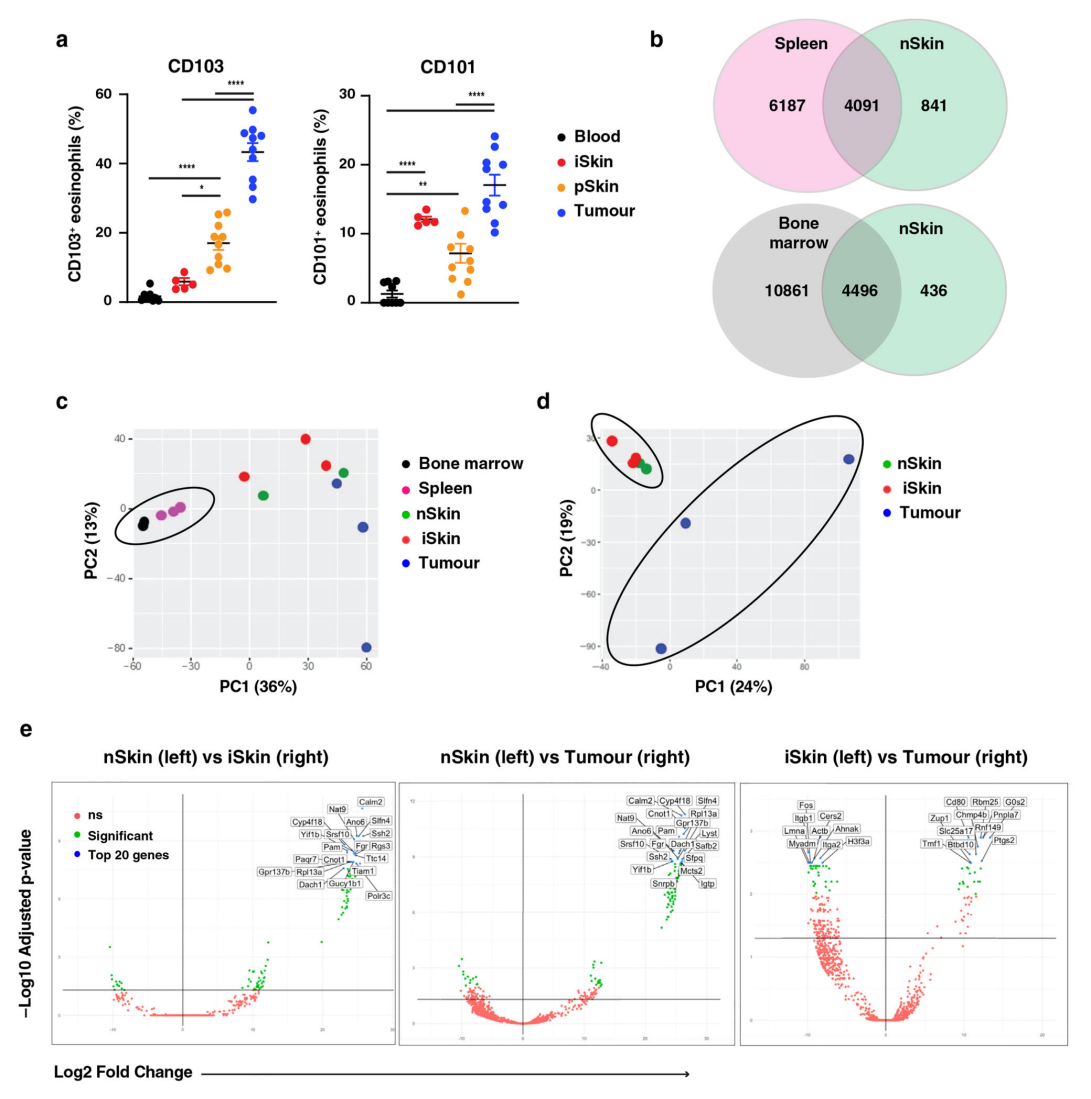
Eosinophils dynamically alter their phenotype according to skin microenvironment. (**a**) Blood and tissue were collected from WT mice, and expressions of CD103 and CD101 on CD45^+^Siglec-F^+^CD11b^+^ eosinophils were analyzed by flow cytometry. iSkin was taken after topical exposure to TPA (2 times per week for 2 weeks), and pSkin and tumors were collected at week 20 after DMBA-TPA carcinogenesis. Data are expressed as mean ± SEM and were analyzed by 1-way ANOVA multiple comparison. *P < .05, ***P < .001, and ****P < .0001. (**b−e**) Eosinophils were FACS sorted as CD45^+^Siglec-F^+^CD11b^+^ live leukocytes from bone marrow (n = 3), spleen (n = 3), nSkin (n = 2), iSkin (n = 3), and tumor (n = 3) of WT mice, and RNA was isolated. Bone marrow, spleen, and nSkin samples were collected from naïve resting mice, iSkin was collected after topical exposure to TPA (2 times per week for 2 weeks), and tumor tissue was collected after DMBA-TPA carcinogenesis. RNA was processed and sequenced using TempO-Seq. (**b**) Venn diagrams showing the numbers of meaningfully expressed genes by eosinophils from naïve bone marrow and spleen versus nSkin. (**c**) Projection of principal components 1 and 2 after application of PCA on all samples after DESeq2 normalization. (**d**) PCA of skin samples only, comparing gene expression by eosinophils from nSkin, iSkin, and tumors. (**e)** Volcano plots showing differentially expressed genes between different skin tissue states with a log fold change thresholds >1 and an adjusted *P* = .05. DMBA, 7,12-dimethylbenz[a]anthracene; iSkin, inflamed skin; ns, not significant; nSkin, naive skin; PC, principal component; PCA, principal component analysis; TPA, 12-O-tetradecanoylphorbol-13-acetate; WT, wild-type.

Although the tissue-infiltrating eosinophils were initially primarily located in the dermis (Figure 1c) and the peritumoral space of cancerous skin (Figure 2c), tissue eosinophils significantly upregulated the expression of the integrin CD103 once in the skin or tumor tissue (Figure 4a). CD103 confers the capacity for tissue retention and interaction with ECs by binding to E-cadherin. Indeed, in the tumors, eosinophils frequently crossed the basement membrane into the epithelium (Figure 2c). Flow cytometry also revealed a significantly altered expression of the surface protein CD101 on tissue eosinophils, compared with that on blood eosinophils, with a particularly high expression on tumor eosinophils (Figure 4a). CD101 has been suggested to be a marker of activated inflammatory eosinophils and to be involved in inhibitory costimulation of T cells (Mesnil et al, 2016; Noble et al, 2024).

To explore whether skin eosinophils might be a unique population, we compared their transcriptome with that of eosinophils from the spleen and the bone marrow. Because eosinophils contain potent ribonucleases, it has proven notoriously difficult to obtain enough good-quality RNA from eosinophils for conventional sequencing. Thus, we utilized a probe-based system (TempO-Seq) that targets the whole genome but is more efficient in preserving partly degraded and/or low-abundance RNA. This analysis showed that although eosinophils in different steady-state tissues clearly expressed a number of core genes in common, there were also many genes that were expressed in a tissue-specific manner (Figure 4b). Principal component analysis revealed that bone marrow and spleen eosinophils were more alike than eosinophils in the skin (Figure 4c). Comparing only skin tissue eosinophils showed that nSkin and iSkin eosinophils had a more comparable gene expression than tumor eosinophils, which were more heterogeneous (Figure 4d). Volcano plot analysis demonstrated that both iSkin eosinophils and tumor eosinophils had gene expression significantly different from that of nSkin eosinophils but also different from each other (Figure 4e and Supplementary Figure S3). Of interest, tumor eosinophils had significantly upregulated expression of *CD80* and *Ptgs2*, with CD80 having been noted on other activated eosinophils and suggesting possible interactions with T cells and *Ptgs* being responsible for biosynthesis of prostaglandins. Together, these data reveal that skin eosinophils display a unique transcriptome that is dynamically altered according to the tissue microenvironment.

### The skin exhibits a distinct lipid mediator profile in healthy, inflamed, and cSCC tissue, and this is regulated by eosinophils

**Figure 5:**
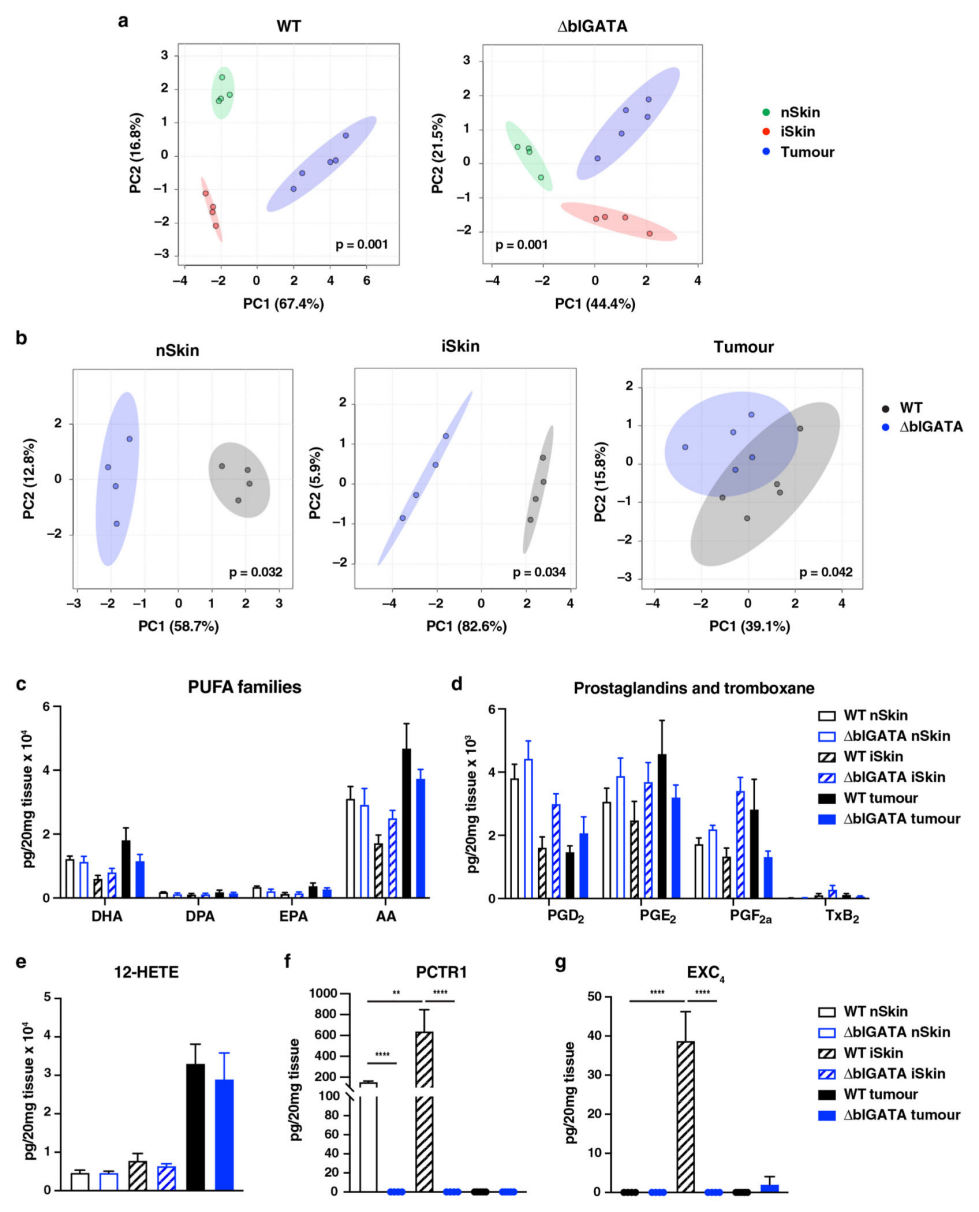
The skin exhibits a distinct lipid mediator profile in healthy, inflamed, and cSCC tissues, and this is regulated by eosinophils. The bioactive lipid mediator profiles of nSkin (n = 4), iSkin (n = 4) (induced by topical TPA), and cSCC tumor (n = 5) tissues (induced by DMBA-TPA carcinogenesis) were analyzed by LC-MS/MS from both WT and ΔdblGATA mice. (**a**) PCA score plots comparing the lipid mediator expression between nSkin, iSkin, and tumor from WT (left plot) and ΔdblGATA mice (right plot). (**b**) PCA score plots showing lipid mediator expression in nSkin, iSkin, and tumor comparing WT with ΔdblGATA mice directly in each tissue state. Ellipses in the score plots denotes 95% confidence regions. Statistical test of the difference between clusters in **a** and **b** was by PERMANOVA. (**c−g**) Expression of (c) PUFA families and (**d−g**) specific bioactive lipid mediators (pg/20mg tissue) in nSkin, iSkin, or tumor tissue from WT and ΔdblGATA mice. Data are expressed as mean ± SEM. Statistics were tested by one-way ANOVA multicomparison. **P < .01 and ****P < .0001. AA, arachnoid acid; cSCC, cutaneous squamous cell carcinoma; DMBA, 7,12-dimethylbenz[a]anthracene; iSkin, inflamed skin; LC-MS/MS, liquid chromatography tandem mass spectrometry; nSkin, naive skin; PCA, principal component analysis; PERMANOVA, permutational multivariate ANOVA; PUFA, polyunsaturated fatty acid; TPA, 12-O-tetradecanoylphorbol-13-acetate; WT, wild-type.

Driven by the discovery that tumor eosinophils upregulate genes for enzymatic oxidation of arachidonic acid and that eosinophils are known producers of lipid mediators, we next explored the skin bioactive lipidome in more detail. Little is currently known of how bioactive lipid mediators contribute to skin homeostasis, inflammation, and cancer, although a plethora of bioactive lipid metabolites are produced from polyunsaturated fatty acids in the skin in response to a variety of stimuli. These are signaling lipids that can have strong effects on tissue health through both skin-resident stromal cells, neuro and immune cells, and skin-infiltrating cells. To investigate their expression, we conducted liquid chromatography tandem mass spectrometry on whole-tissue biopsies from nSkin, iSkin, and tumor tissue. We found that the bioactive lipid mediators expressed in the skin were significantly altered in inflammation and in cSCC (Figure 5a). This change in the bioactive lipidome in inflamed and cancerous tissue was true in both WT and eosinophil-deficient mice (Figure 5a); however, the profile of the metabolites expressed were significantly impacted by the presence of eosinophils both in nSkin, iSkin, and tumor (Figure 5b) (the variance explained by the principal components are shown in Supplementary Figure S4a and b).

A whole range of both proinflammatory and proresolving lipid mediators was present in the tissue, including from the families of leukotrienes, prostaglandins, hydroxyeicosatetraenoic acids, resolvins, protectins, maresins, lipoxins, and eoxins. These lipid mediators are synthesized predominantly by enzymatic oxidation of arachidonic acid and docosahexaenoic acid, and indeed, these 3 families of polyunsaturated fatty acid were the most dominantly expressed in all tissue states, with no difference in whether eosinophils were present or not (Figure 5c). Of their products, most wellstudied are the prostanoid lipid mediators synthesized by COX; however, although we detected most of the COX-derived prostanoids in good quantity in the skin, they were not significantly raised during inflammation or in the tumor tissue, and there was no difference between WT and ΔdblGATA mice (Figure 5d). What was most notable in the tumor tissue was a strong expression of lipid mediators metabolized by the LOX pathway. For example, the hydroxyeicosatetraenoic acids and, particularly, 12-hydroxyeicosatetraenoic acid, which is considered to be a potent proinflammatory mediator, were very highly and specifically expressed in the tumor tissue, albeit this was not regulated by the presence of eosinophils (Figure 5e). However, we detected some strong effects on other LOX-generated lipid mediator families such as the protectins and eoxins, the most abundant being PCTR1 and EXC_4_, both of which were upregulated in iSkin of WT mice but completely absent in mice lacking eosinophils and also not present in tumor tissue (Figure 5f and g and representative chromatograms shown in Supplementary Figure S4c and d). Together, our data demonstrate a dynamic regulation of lipid mediators in healthy and diseased skin tissue and that the presence of eosinophils in the skin affect the bioactive lipidome, particularly lipid mediators generated by the LOX pathways.

### PCTR1 regulates epithelial turnover and epidermal hyperplasia and can limit cSCC growth

**Figure 6:**
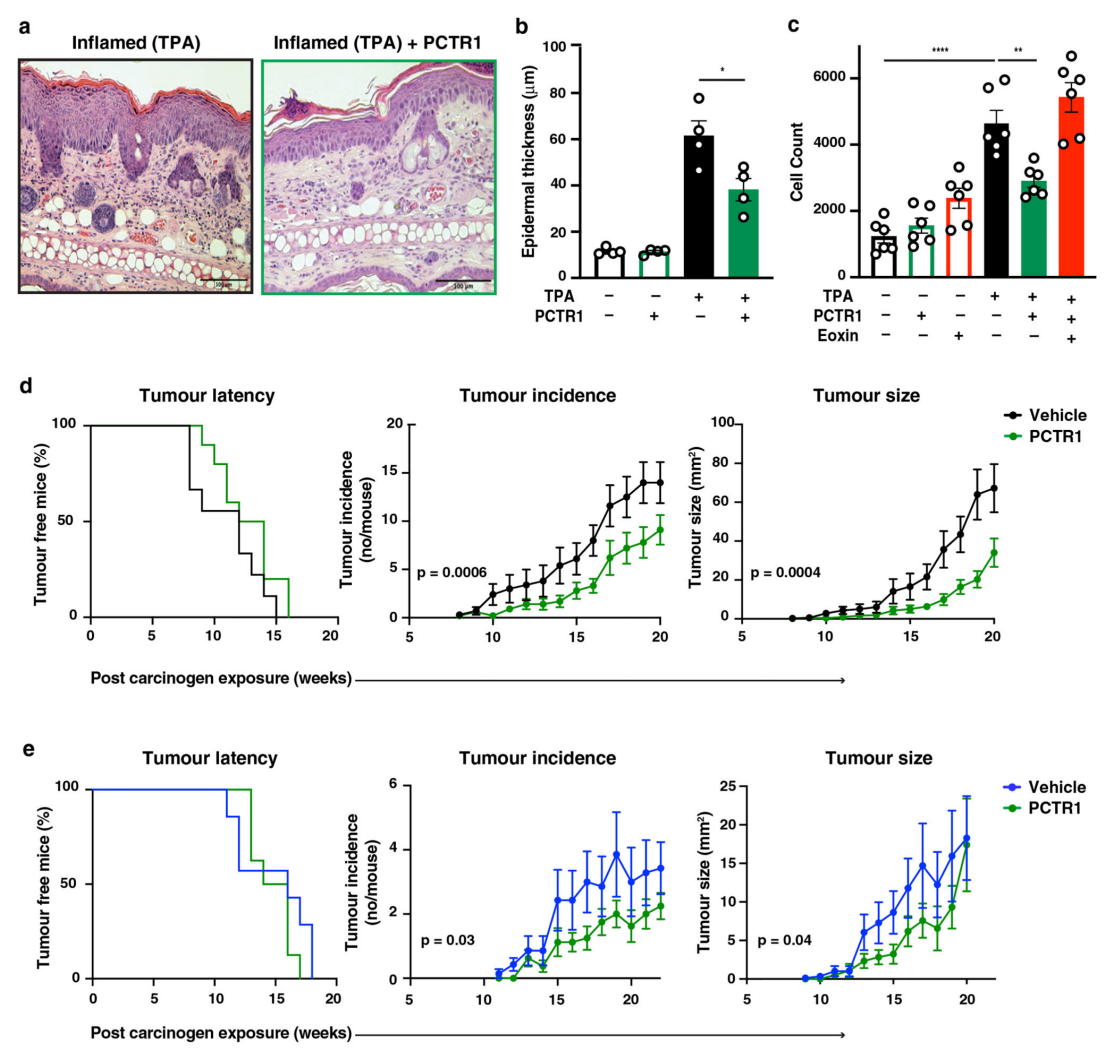
PCTR1 regulates epithelial turnover and epidermal hyperplasia and can limit cSCC growth. (**a, b**) The dorsal ear skin of WT and ΔdblGATA mice was treated with either ethanol (vehicle control), PCTR1, TPA, or TPA + PCTR1 twice weekly over 2 weeks. (**a**) Representative images of H&E-stained cross-sections of iSkin with/without PCTR1 treatment (bar = 100 μm) and (b) epidermal thickness as measured using Fiji software (n = 4). (**c**) The cSCC cell line PAM212 was grown in vitro with/without the inflammatory agent TPA and with/without the addition of PCTR1 and EXC_4_, and cells were counted after 72 h in culture (n = 6 per group). (**d**) WT (n = 10 per group) and (**e**) ΔdblGATA (n = 8 per group) mice underwent DMBA-TPA inflammation-driven carcinogenesis with/without additional topical PCTR1 treatment twice a week. Tumor susceptibility is expressed as tumor latency (time to appearance of first tumor), tumor incidence (average number of tumors per mouse), and tumor area (average tumor size per mouse). Data are expressed as mean ± SEM, with statistics tested by 1-way ANOVA multiple comparison (for b and c) and log-rank (Mantel-Cox) test for tumor latency and linear regression for tumor incidence and area (for **d** and **e**). *P < .05, **P < .01, and ****P < .0001. cSCC, cutaneous squamous cell carcinoma; DMBA, 7,12-dimethylbenz[a]anthracene; h, hour; iSkin, inflamed skin; TPA, 12-O-tetradecanoylphorbol-13-acetate; WT, wild-type.

Eoxins are thought to be produced mostly by eosinophils (with some contribution from mast cells) and to have strongly proinflammatory properties, whereas protectin conjugates in tissue regeneration are proresolving mediators that may be produced by eosinophils themselves but can also be produced by other cell types, including macrophages (Feltenmark et al, 2008; Ramon et al, 2016). Because we found that PCTR1 and EXC_4_ were highly expressed in iSkin of WT mice and that eosinophils drove the tumor-promoting inflammation, we hypothesized that eosinophil-driven proinflammatory mediators leads to production of tissue repair mediators. Next, we tested the effect of PCTR1 and EXC_4_ in the skin both in vivo and in vitro. We found in vivo that simply adding PCTR1 topically to inflamed skin markedly reduced epidermal hyperplasia (Figure 6a and b). To test whether this could be a direct effect on the ECs, we grew the cSCC cell line PAM212 in vitro and then added PCTR1 and looked at effects on cell proliferation. This showed that PCTR1 reduced the inflammation-driven expansion of the tumor cells and, furthermore, that this effect was abolished by the addition of EXC_4_, which allowed for continued tumor cell expansion (Figure 6c). Addition of PCTR1 or EXC_4_ alone did not have a significant effect on PAM212 expansion in this context. To explore whether PCTR1 could also regulate cancer susceptibility de novo, we examined its effect on the inflammation-driven model of cSCC (DMBA-TPA). This showed that mice treated topically with PCTR1 twice a week had a significantly reduced incidence of tumors and also developed smaller tumors than vehicletreated mice, although there was no significant difference in latency (Figure 6d). Addition of PCTR1 to the skin of eosinophil-deficient mice during carcinogenesis showed a similar effect of reduced number and size of tumors, albeit these mice already had a reduced tumor susceptibility compared with WT mice (Figure 6e). The addition of EXC_4_ had no effects at the dose tested (Supplementary Figure S5). Thus, lipid mediators can regulate EC expansion in response to inflammation, and the anti-inflammatory mediator PCTR1 can reduce tumor growth.

## Discussion

In this study, we found that eosinophils rapidly infiltrated inflamed skin in a CCR3-dependent manner and accumulated in cSCC tissue. In the inflamed skin, they displayed a unique phenotype and induced the expression of particular LOX-derived lipid mediators. Consequently, the presence of eosinophils during skin inflammation enhanced EC turnover and epidermal hyperplasia and promoted the outgrowth of tumors. We further report that topical treatment with the lipid mediator PCTR1 reduced epithelial expansion and slowed tumor growth.

Eosinophils are often found at tissue sites undergoing high EC turnover or remodeling, and it is well-established that eosinophils predominately use CCR3 to traffic into other tissues such as the lung (Fulkerson et al, 2006; Humbles et al, 2002). In this study, we show that eosinophils are present with low abundance in healthy resting skin, but they rapidly accumulate during inflammation-induced damage and remodeling, suggesting that they may be inherently involved in regulating these processes. Indeed, we found that the absence of eosinophils significantly reduced epidermal hyperplasia during an inflammatory insult and particularly limited the expansion of basal ECs. This is in line with findings from other tissues where the absence of eosinophils in the intestinal tissue was shown to reduce EC turnover and villous surface area (Ignacio et al, 2022) and in the thymus to reduce EC numbers and thymus size after irradiation (Cosway et al, 2022).

Although healthy skin is home to relatively few eosinophils, and we did not find any gross morphological tissue differences in their absence, our data showed that eosinophils do influence the skin even at steady state. We found a significant shift in the expression of bioactive lipid mediators in the skin in the absence of eosinophils even in healthy resting skin, suggesting that eosinophils may also exert subtle activities in the tissue during health, as have been shown in tissues with a larger homeostatic eosinophil population (Gurtner et al, 2023).

It is now appreciated that eosinophils can exhibit phenotypic diversity, and indeed, our data support the notion that eosinophils are highly plastic in nature. We show that eosinophils have adapted to the skin niche and exhibit a distinct transcriptional profile compared with bone marrow and spleen eosinophils but also that local cues in the skin during different tissue challenges induced specific properties and phenotypic diversity in the eosinophils. Diversity of this kind has been demonstrated previously in the intestine, where transcriptomic profiling has identified a basal and an active population of eosinophils (Gurtner et al, 2023). Two distinct subpopulations of eosinophils have also been identified in the asthmatic lung on the basis of their expression of CD101, with tissue-resident eosinophils being CD101^lo^, whereas recruited eosinophils were CD101^hi^ and exhibited an inflammatory profile (Mesnil et al, 2016). We similarly showed a marked higher expression of CD101 on eosinophils in iSkin and in tumor tissue than on resting skin eosinophils, and overall, our transcriptomic and phenotyping data support the notion of tissue adaptation, plasticity, and functional specialization in the eosinophil lineage.

Although tissue-associated tumor eosinophilia has been noted in several solid tumors previously, it is much less clear whether and how they contribute to the TME and tumor growth regulation. Both pro and antitumor functions of eosinophils have been documented in experimental models (Grisaru-Tal et al, 2020), and indeed, the role of eosinophils may be cancer specific owing to their dynamic adaptation to different tissues. In this study, we show unequivocally that eosinophils promote epithelial expansion in the skin, enhancing inflammation-driven cSCC growth. A similar protumorigenic role for eosinophils has been described in several models of carcinogen-induced oral SCC (Wong et al, 1999). Eosinophils could promote tumor growth in numerous ways; they store and release many growth factors and cytokines in abundance such as, for example, VEGF, epidermal growth factor, TGFb, and IL-4 (Grisaru-Tal et al, 2020). Eosinophil-derived TGFb has been linked to epithelial growth and remodeling (Yasukawa et al, 2013), and eosinophils have been shown to promote angiogenesis (Puxeddu et al, 2005). We have also previously shown that IL-4 is a potent driver of inflammation-driven cSCC (Dalessandri et al, 2016). Eosinophils could influence tumor growth by at least 2 non-mutually exclusive mechanisms: direct or indirect interaction with mutated tumor cells or by cross-regulation of other leukocytes in the TME. Although we did not find any difference in the level of tissue immune infiltrate or the relative distribution of immune cell subsets or their phenotype in the TME in the absence of eosinophils, we did not exhaustively test whether eosinophils may influence other immune cells in the tumor tissue or whether any such indirect effects could influence tumor growth. Our data indeed indicated that tumor eosinophils upregulated the expression of T-cell costimulatory molecules, such as CD101 and CD80, suggesting influences on T-cell function in the TME. However, we most notably saw effects on ECs and showed that eosinophildriven bioactive lipid mediators could directly regulate tumor cell growth in vitro.

There has been substantial interest in bioactive lipid mediators for treatment of cancer. The effort has thus far been particularly focussed on eicosanoid mediators metabolized by COX. Notably, a large body of evidence suggests that regular use of aspirin or other nonsteroidal anti-inflammatory drugs (which inhibit COX) is associated with reduced risk of developing a number of epithelial cancers (Cuzick et al, 2009; Langley et al, 2011). However, concerns about toxicity have limited the use of aspirin as a cancer-prevention agent, and other strategies are being sought. We found that inflamed skin and cSCC tissue are particularly dominated by lipid mediators synthesized by LOX enzymes and that eosinophils in the tissue predominantly regulated 15-LOX–derived lipid mediators. It has previously been shown that LOX activity is an important mediator of skin inflammation, and an imbalance in LOX enzyme activity has been suggested to be crucial for disease etiology in psoriasis (Kendall and Nicolaou, 2013; Simard et al, 2022). Concurrently, a paradigm shift is emerging in understanding the resolution of inflammation as an active biochemical process with the discovery of specialized proresolving anti-inflammatory lipid mediators such as the eicosanoid families of resolvins, protectins, and maresins (Serhan and Levy, 2018). Our data demonstrate that eosinophils regulate both potent proinflammatory (eoxins) and proresolving (PCTR) LOX-derived mediators in the skin. We hypothesize a temporal control of these LOX-derived lipid mediators and that the lack of eosinophils and their proinflammatory products, such as eoxins, consequently lowers the production of PCTR1 in the skin. Our data further demonstrate the ability of PCTR1 to reduce epithelial hyperplasia and tumor growth both in vivo and in vitro. It is likely that PCTR1 predominantly acts at the stage of tumor cell expansion and progression rather than during the actual process of carcinogenesis, because we did not observe an effect of PCTR1 on tumor latency. Indeed, some other recent work has started to assess whether stimulating the resolution of inflammation using proresolving lipid mediators can inhibit cancer progression, and promising data using PCTR1 in breast cancer show a sharp reduction in growth both when used alone and in combination with immunotherapy (a-CTLA4) (Kipper et al, 2022).

Together, our findings demonstrate that eosinophils and specific bioactive lipid mediators play important roles in skin biology and EC carcinogenesis and point to potential novel therapeutic strategies for both chronic inflammatory disorders and epithelial malignancies.

## Materials and Methods

### Mice

ΔdblGATA mice were generated as previously described (Yu et al, 2002) and used on the BALB/c background. BALB/c WT mice were purchased from Charles River. All mice were bred and maintained in individually ventilated cages under specific pathogen-free conditions, with food and water provided ad libitum. Age-matched, female mice were used for all experiments at the age ≥7 weeks and selected at random from a large pool when allocated to experiments. All studies complied with Imperial College AWERB (Animal Welfare and Ethical Review) guidelines and UK Home Office regulations and were approved by Imperial College and the UK Home Office. All experiments involving cancer growth strictly adhered to the guidelines set out by the National Cancer Research Institute and Workman et al (2010) in ‘Guidelines for the Welfare and Use of Animals in Cancer Research.’ All studies using animals were conducted following the Animal Research: Reporting In Vivo Experiments guidelines (Percie du Sert et al, 2020).

### Skin challenge and cutaneous carcinogenesis

The chemicals TPA and DMBA were purchased from Sigma and dissolved in 100% ethanol and acetone, respectively. PCTR1 and EXC_4_ were purchased from Cayman Chemical and dissolved in 100% ethanol, and concentration and purity were confirmed by mass spectrometry.

For cutaneous carcinogenesis, age-matched female mice were used at the age of 7 weeks. The dorsal back fur was shaved with clippers, and mice rested for 1 week. Applications of chemicals and tumor monitoring were performed as previously described (Strid et al, 2008). In brief, 600 nM DMBA was carefully applied to the entire shaved skin area in a volume of 150 µl. Mice were rested for 1 week, and 20 nM TPA was then applied twice weekly on the entire back skin. For tumor experiments, including lipid mediators, 770 nM PCTR1 in 100 µl was additionally applied twice weekly on the entire back skin. Hair regrowth during the experiment was gently removed using hair clippers. Mice were monitored twice weekly with clinical scores, and tumors were measured by callipers once a week.

Acute skin inflammation was promoted by repeated topical application of 12.5 µl of 2.5 nM TPA in 25 µl to the dorsal side of the ear skin twice weekly for 2 weeks. For experiments including lipid mediators, 1500 nM PCTR1 was additionally added twice a week.

### Cell culture

PAM212 and CT26 cell lines were purchased from ATCC and maintained in RPMI 1640 GlutaMax medium (Gibco) supplemented with 10% fetal bovine serum and 1% penicillin–estreptomycin–eglutamine (Gibco) at 37 °C.

For PAM212 cell stimulation and quantification, PAM212 cells were seeded at a density of 4 × 10^4^ cells per well in 12-well plates. Cells were treated with 20 ng/ml TPA, 5 nM PCTR1, and/or 5 nM EXC_4_ (Cayman Chemical) for 72 hours. Cells were subsequently stained with NucBlueTM Live Cell Stain (Invitrogen) and visualized by Leica TCS SP5 confocal microscopy (Leica Microsystems). A ‘4 × 4’ tile scan compromising 16 fields was performed at the center of each well. Cell quantification was performed by automated cell nuclei counting function with Fiji ImageJ software (version 1.53s).

### Subcutaneous tumor inoculation

CT26 cells were used at 75% confluency. Prior to injection, the right flanks of mice were shaved using clippers, and 1 × 10^5^ CT26 cells were injected subcutaneously in PBS. Tumor growth was monitored and measured with callipers daily, and the experiment was terminated when tumors reached 12.5 mm in diameter.

### CCR3 blockade

BALB/c WT mice were injected intravenously with 3 µg of anti-CCR3 antibody (clone: 83103, R&D Systems) or rat antimouse IgG2A isotype antibody control (clone R19-15, BD Pharmingen) followed by a single topical exposure of 30 µl of 5.7 nM TPA on the dorsal ear skin. Six hours later, mice received a second anti-CCR3 injection. Skin was collected 18 hours later for further analysis.

### Circulating CD45^+^ cell labeling

After TPA ear skin challenge twice over a week, BALB/c WT mice were injected intravenously with 5 µg anti-CD45 allophycocyanin (clone: 30-F11, eBioscience) and culled 1 minute later by cervical dislocation. Blood and skin were immediately collected and further processed for flow cytometry.

### Tissue processing

For single-cell suspensions, mouse tissue was cut into small 1-mm^2^ pieces using a scalpel blade and incubated for 1.5 hours in digestion buffer containing 25 µg/ml Liberase (Roche), 250 mg/ml DNAse l (Roche), and 1 × DNAse buffer (1.21 g Tris Base, 0.5 g magnesium chloride, and 0.073 g calcium chloride made up to 100 ml with double-distilled water and pH adjusted to 7.5) in PBS at 37 °C. Tissue was then transferred to C-tubes (Miltenyi Biotech) containing complete RPMI-1640 medium and dissociated using a gentleMACS Dissociator (Miltenyi Biotech). Cell suspensions were then filtered through a 70-µl strainer and counted using a CASY cell counter (Roche).

For blood, venous blood was collected from the tail vein and was transferred to 100 mM EDTA and stained with appropriate antibodies for flow cytometry. Cells were then incubated for 15 minutes with FACS lysis buffer (BD Bioscience) for red cell lysis and subsequently washed in PBS.

### Flow cytometry

Single-cell suspensions were blocked for nonspecific binding using antimouse CD16/32 TruStain FcX (BioLegend) and 2% normal rat serum (Sigma) prior to any staining protocols. For staining of cell surface markers, fluorochrome-conjugated antibodies and live/dead discrimination dye (Invitrogen) were added to cell suspensions for 25 minutes and subsequently washed. For intranuclear FoxP3 staining, the FoxP3 staining kit (Thermo Fisher Scientific) was used as per the manufacturer’s instructions. Stained cells were analyzed using BD FACSVerse and Fortessa X20 (BD Biosciences) machines. Data analysis was performed using FlowJo 10.6.1 for Mac (TreeStar). For eosinophil cell sorting, a FACS Aria III High Speed Cell Sorter (BD Bioscience) was used.

Antibodies were sourced from BioLegend unless otherwise stated. The following antibodies were used: anti-CD45 (30-F11, eBioscience), anti-CD11b (M1/70), anti-Siglec-F (E50-2440), anti-Ly6C (HK1.4), anti-Ly6G (IA8), anti-CD4 (GK1.5), anti-CD8 (53e6.7), anti-IgE (23G3, eBioscience), anti-TCRb (H57-597), anti-TCRgd (eBioGL3, eBioscience), anti-CD117 (2B8), anti-CD103 (2E7), anti-CD101 (Moushi101, Invitrogen), anti-CCR3 (J073E5), anti-CD11c (HL3, BD Pharmingen), anti-FoxP3 (FJK-165, Invitrogen), anti-NKG2D (CX5, BD Horizon), anti-B220 (RA3-6B2), and CD127 (eBioSB/199).

Cell subsets were gated on live singlets for all and then for eosinophils (CD45^+^GR1^+^CD11b^+^Siglec-F^+^), neutrophils (CD45^+^ IgE^-^Siglec-F^-^Ly6C^mid^Ly6G^+^), monocytes (CD45^+^CD11b^+^Ly6G^e^ Ly6C^+^), macrophages (CD45^+^IgE^-^Siglec-F^-^Ly6C^-^Ly6G^-^F4/80^+^), αβ T cells (CD45^+^Tcrb^+^CD4/CD8^+^), γδ T cells (CD45^+^Tcrd^+^), mast cells (CD45^+^IgE^+^CD117^+^), basophils (CD45^mid^IgE^+^CD117 ^−^), ILC2 (CD45^+^LineageNeg [excluding CD2, CD3, CD4, CD8, CD11b, CD11c, FcεR1, Langerin, TCRβ, and TCRd] and CD127^+^CD103^+^), dendritic cells (CD45^+^IgE^-^Siglec-F^-^Ly6C^-^Ly6G^-^F4/80^-^CD11c^−^), regulatory T cell (CD45^+^Tcrb^+^CD8^-^CD4^+^FoxP3^+^), B cells (CD45^+^Tcrb^-^CD11b^-^TCRd^-^CD19^+^), and NK cells (CD45^+^ Tcrb^-^CD11b^-^TCRd^-^NKG2D^+^).

### Epidermal thickness

Whole-mouse ear skin was fixed and paraffin embedded in parallel. A total of 5-μm sections were cut and stained for H&E. Images were obtained using a Leica DM4B microscope (Leica Biosystems), and epidermal thickness was measured using Fiji software by taking 7 measurements per section from 7 sections per ear in a blinded manner.

### Immunofluorescence

Murine tissue was snap frozen in optimal cutting temperature on dry ice. A total of 8-μm sections were cut using a Leica JUNG CM1800 cryostat and stored at -80 °C. For staining, slides were returned to room temperature and fixed with 4% paraformaldehyde for 15 minutes. Samples were then blocked in 5% goat serum (Sigma) for 1 hour in room temperature and stained with anti-Siglec-F (IRNM44N, Invitrogen) overnight at 4 °C. After staining, samples were washed and incubated with Alexa Fluor 555–conjugated goat antirat IgG (A21434, Thermo Fisher Scientific) and then further incubated with Alexa Fluor 647econjugated rat antimouse CD49F (GoH3, Bio-Legend). Sections were then directly mounted in VectaShield containing DAPI (Vectashield). Tissue samples were visualized with a Leica SP5 confocal laser-scanning microscope (Leica).

### RT-qPCR and primer sequences

Murine ears were collected and split into dorsal and ventral sides, and the dorsal side was floated epidermal side up in NH4SCN (Sigma-Aldrich) for 40 minutes at 37 °C, and the epidermis was separated from the dermis using forceps and preserved in RNA-later. Total tissue RNA was extracted using RNEasy Mini kits (Qiagen). RNA was then dissolved in nuclease-free water, and yield and purity were determined. cDNA was synthesized from RNA with iScript cDNA synthesis kit (Bio-Rad Laboratories), as per the manufacturer’s instructions. cDNA was diluted in nuclease-free double-deionized water for RT-qPCR. All primers were single-stranded DNA oligonucleotides (Sigma) that were intron spanning as verified by National Center for Biotechnology Information Primer-Blast tool. Real-Time PCR products were detected with SYBR Green (Thermo Fisher Scientific) measured continuously with a ViiA 7 Real-Time PCR system (Applied Biosystems). Ct values for genes of interest were normalized to products of amplification of the housekeeping gene cyclophilin, and relative expression was calculated using the 2-ΔCt method. The following primers were used: keratin 1: forward 5´-TTTGCCTCCTTCATCGACA-3´ and reverse 5´-GTTTTGGGTCC GGGTTGT-3´; keratin 5: forward 5´-CCTGCAGAAGGCCAAGCA-3´ and reverse 5´-TGGTGTTCATGAGCTCCTGGTA-3´; keratin 10: forward 5´-GGATGAGCTGACCCTTAGCA-3´ and reverse 5´-CATTTTGAAGGTCTCTCATTTCCT-3´; keratin 14: forward 5´-CAGCCCCTAC TTCAAGACCA-3´ and reverse 5´-GGCTCTCAATCTGCATCTCC-3´; and cyclophilin: forward 5´-CAAATGCTGGACCAAACACAA-3´ and reverse 5´-CCATCCAGCCATTCAGTCTTG-3´.

### Human tissue

The study of human tissue was conducted according to the Declaration of Helsinki Principles, and all patients donating samples to this study provided written, informed consent in accordance with ethical approval. All tumor tissue used in the study was confirmed cSCC, and the tumor perilesional skin, excised as ‘dog ears’ adjacent to the cSCC, was histopathologically normal. For cSCC and tumor perilesional tissue, research ethics number was (REC NHS REC 08.S1401.69). Healthy skin was collected as surplus skin from elective surgical procedures (eg, breast or abdominal reductive surgery) in collaboration with the Imperial College Healthcare Tissue Bank with Research Ethics Committee approval number 22/WA/0214.

For normal healthy skin, the subcutaneous fat was removed using scissors prior to further processing. To obtain single-cell suspension, healthy skin or tumor tissue was cut into small pieces and was enzymatically digested for 2 hours with 10 μg/ml DNAse l (Roche) and 1 mg/ml collagenase IA (Sigma) in PBS shaking at 37 °C. Tissue was then strained through a 70-μm cell strainer into complete RPMI-1640 medium. Single-cell suspensions were stained for FACS against CD45 (2D1, BioLegend), CD14 (M5E2, BioLegend), CD117 (104D2, BioLegend), CD11b (ICRF44, BD Biosciences), and Siglec-8 (837535, BD Biosciences). Cells were visualized using Cytek Aurora (Cytek), and data analysis was performed using FlowJo 10.6.1 for Mac. Eosinophils were gated on live singlets cells and then as CD45^+^CD14^-^CD117^-^CD11b^+^Siglec-8^+^.

For immunohistochemistry of cSCC tissue, 5-μm tissue sections were cut from formalin-fixed, paraffin-embedded samples and placed onto 3-aminopropyltriethoxysilaneecoated microscope slides. Sections were deparaffinized in xylene and rehydrated through graded alcohols. Antigen retrieval was performed by heating sections under pressure in citrate buffer pH 6, and endogenous peroxidase was inhibited with 3% hydrogen peroxide in PBS. Nonspecific binding was blocked by incubating sections in 10% foetal bovine serum (Gibco) and 1% BSA (Sigma) for 30 minutes at room temperature. The anti-Ribonuclease 3/eosinophil cationic protein antibody (EPR20357, Abcam) was then incubated at 4 °C for 16 hours before 3 Tris buffered saline washes. Slides were then incubated with horseradish peroxidaseeconjugated secondary antibody (Thermo Fisher Scientific) and visualized using 3,3’-diaminobenzidine (Sigma). After washing with Tris buffered saline, sections were counterstained with Gill’s hematoxylin (Sigma), and slides were subsequently dehydrated through graded alcohols and xylene. Images were obtained using the Aperio CS2 Scanner at ×40 magnification and analyzed with Aperio ImageScope ×64 (version 12.4.3.5008).

### Lipid mediator quantification

Skin tissue was collected, snap frozen on dry ice, and maintained at -80 °C until processing for liquid chromatography tandem mass spectrometry analysis. Lipid mediators were identified and quantified as previously described (Gomez et al, 2024; Koenis et al, 2024) and detailed in the Supplementary Materials and Methods. Data analysis was performed using MetaboAnalyst software, version 6.0 (Pang et al, 2024). The *P*-values for principal component analysis plots were calculated using permutational multivariate ANOVA.

### Transcriptomics

FACS-sorted eosinophils were resuspended in RLT buffer (Qiagen) supplemented with 1% β-mercaptoethanol (Sigma-Aldrich), and RNA was extracted using RNEase Micro kits (Qiagen). Transcriptomics analysis was performed using the TempO-Seq whole-genome coding transcriptome platform with a proprietary processing pipeline (Bioclavis) (Yeakley et al, 2017). Normalization of read counts was performed using DESeq2 in R studio 4.1.1 (RStudio). Differential gene expression was performed by pairwise comparisons with adjusted *P* < .05 and log_2_ (F=fold change) ≥1 (corresponding to a 2-fold difference in gene expression).

### Statistical analysis

The statistical significance of difference between groups was performed using 2-tailed Student’s *t*-test for unpaired data and 1-way ANOVA or linear regression, where appropriate, with the results deemed significant at *P* < .05. Stars of significance correlate to *P < .05, **P < .01, ***P < .001, and ****P < .0001. Statistics was performed with GraphPad Prism 8 for Mac (GraphPad). Error bars represent the SEM.

## Supplementary Material

Supplementary material is linked to the online version of the paper at www.jidonline.org, and at https://doi.org/10.1016/j.jid.2025.04.015.

Fig. S1

Fig. S2

Fig. S3

Fig. S4

Fig. S5

Supplementary text

## Data Availability

The data supporting the findings of this study are available from the corresponding author upon request. The raw liquid chromatography tandem mass spectrometry data are available from the public repository at BioStudies (accession number S-BSST1722) https://www.ebi.ac.uk/biostudies/studies?query=S-BSST1722.

## Abbreviations

COX: cyclooxygenase
cSCC: cutaneous squamous cell carcinoma
DMBA: 7,12-dimethylbenz[a]anthracene
EC: epithelial cell
iSkin: inflamed skin
LOX: lipoxygenase
nSkin: naive skin
TME: tumor microenvironment
TPA: 12-O-tetradecanoylphorbol-13-acetate
WT: wild-type

## References

[R1] Abel EL, Angel JM, Kiguchi K, DiGiovanni J (2009). Multi-stage chemical carcino-genesis in mouse skin: fundamentals and applications. Nat Protoc.

[R2] Cosway EJ, White AJ, Parnell SM, Schweighoffer E, Jolin HE, Bacon A (2022). Eosinophils are an essential element of a type 2 immune axis that controls thymus regeneration. Sci Immunol.

[R3] Coussens LM, Zitvogel L, Palucka AK (2013). Neutralizing tumor-promoting chronic inflammation: a magic bullet?. Science.

[R4] Crawford G, Hayes MD, Seoane RC, Ward S, Dalessandri T, Lai C (2018). Epithelial damage and tissue γδ T cells promote a unique tumor-protective IgE response. Nat Immunol.

[R5] Cuzick J, Otto F, Baron JA, Brown PH, Burn J, Greenwald P (2009). Aspirin and non-steroidal anti-inflammatory drugs for cancer prevention: an international consensus statement. Lancet Oncol.

[R6] Dalessandri T, Crawford G, Hayes M, Castro Seoane R, Strid J (2016). IL-13 from intraepithelial lymphocytes regulates tissue homeostasis and protects against carcinogenesis in the skin. Nat Commun.

[R7] Dalessandri T, Strid J (2014). Beneficial autoimmunity at body surfaces - immune surveillance and rapid type 2 immunity regulate tissue homeostasis and cancer. Front Immunol.

[R8] Diakos CI, Charles KA, McMillan DC, Clarke SJ (2014). Cancer-related inflammation and treatment effectiveness. Lancet Oncol.

[R9] Eggermont CJ, Eggermont AMM (2024). Shifting landscape in skin cancer incidence: the rising tide of cutaneous squamous cell carcinoma and potential implications for prevention. Br J Dermatol.

[R10] Feltenmark S, Gautam N, Brunnström A, Griffiths W, Backman L, Edenius C (2008). Eoxins are proinflammatory arachidonic acid metabolites produced via the 15-lipoxygenase-1 pathway in human eosinophils and mast cells. Proc Natl Acad Sci USA.

[R11] Fulkerson PC, Fischetti CA, McBride ML, Hassman LM, Hogan SP, Rothenberg ME (2006). A central regulatory role for eosinophils and the eotaxin/CCR3 axis in chronic experimental allergic airway inflammation. Proc Natl Acad Sci USA.

[R12] Gomez EA, De Matteis R, Udomjarumanee P, Munroe PB, Dalli J (2024). An LGR6 frameshift variant abrogates receptor expression on select leukocyte subsets and is associated with viral infections. Blood.

[R13] Grisaru-Tal S, Itan M, Klion AD, Munitz A (2020). A new dawn for eosinophils in the tumour microenvironment. Nat Rev Cancer.

[R14] Gurtner A, Crepaz D, Arnold IC (2023). Emerging functions of tissue-resident eosinophils. J Exp Med.

[R15] Harwood CA, Mesher D, McGregor JM, Mitchell L, Leedham-Green M, Raftery M (2013). A surveillance model for skin cancer in organ transplant recipients: a 22-year prospective study in an ethnically diverse population. Am J Transplant.

[R16] Harwood CA, Proby CM, Inman GJ, Leigh IM (2016). The promise of genomics and the development of targeted therapies for cutaneous squamous cell carcinoma. Acta Derm Venereol.

[R17] Humbles AA, Lu B, Friend DS, Okinaga S, Lora J, Al-Garawi A (2002). The murine CCR3 receptor regulates both the role of eosinophils and mast cells in allergen-induced airway inflammation and hyperresponsiveness. Proc Natl Acad Sci USA.

[R18] Ignacio A, Shah K, Bernier-Latmani J, Köller Y, Coakley G, Moyat M (2022). Small intestinal resident eosinophils maintain gut homeostasis following microbial colonization. Immunity.

[R19] Karia PS, Han J, Schmults CD (2013). Cutaneous squamous cell carcinoma: estimated incidence of disease, nodal metastasis, and deaths from disease in the United States, 2012. J Am Acad Dermatol.

[R20] Kendall AC, Nicolaou A (2013). Bioactive lipid mediators in skin inflammation and immunity. Prog Lipid Res.

[R21] Kipper FC, Kelly A, Rothenberger E, Duncan M, Serhan CN, Panigrahy D (2022). Preotectins inhibit receptor negative breast cancer growth in mice. FASEB J.

[R22] Koenis DS, de Matteis R, Rajeeve V, Cutillas P, Dalli J (2024). Efferocyte-derived MCTRs metabolically prime macrophages for continual efferocytosis via Rac1-mediated activation of glycolysis. Adv Sci (Weinh).

[R23] Langley RE, Burdett S, Tierney JF, Cafferty F, Parmar MK, Venning G (2011). Aspirin and cancer: has aspirin been overlooked as an adjuvant therapy?. Br J Cancer.

[R24] Long H, Zhang G, Wang L, Lu Q (2016). Eosinophilic skin diseases: a comprehensive review. Clin Rev Allergy Immunol.

[R25] Mantovani A, Allavena P, Sica A, Balkwill F (2008). Cancer-related inflammation. Nature.

[R26] Martincorena I, Roshan A, Gerstung M, Ellis P, Van Loo P, McLaren S (2015). Tumor evolution. High burden and pervasive positive selection of somatic mutations in normal human skin. Science.

[R27] Marx J (2004). Cancer research. Inflammation and cancer: the link grows stronger. Science.

[R28] Mesnil C, Raulier S, Paulissen G, Xiao X, Birrell MA, Pirottin D (2016). Lung-resident eosinophils represent a distinct regulatory eosinophil subset. J Clin Invest.

[R29] Migden MR, Rischin D, Schmults CD, Guminski A, Hauschild A, Lewis KD (2018). PD-1 blockade with cemiplimab in advanced cutaneous squamous-cell carcinoma. N Engl J Med.

[R30] Nagarajan P, Asgari MM, Green AC, Guhan SM, Arron ST, Proby CM (2019). Keratinocyte carcinomas: current concepts and future research priorities. Clin Cancer Res.

[R31] Noble SL, Vacca F, Hilligan KL, Mules TC, Le Gros G, Inns S (2024). Helminth infection induces a distinct subset of CD101hi lung tissue-infiltrating eosinophils that are differentially regulated by type 2 cytokines. Immunol Cell Biol.

[R32] Pang Z, Lu Y, Zhou G, Hui F, Xu L, Viau C (2024). MetaboAnalyst 6.0: towards a unified platform for metabolomics data processing, analysis and interpretation. Nucleic Acid Res.

[R33] Percie du Sert N, Hurst V, Ahluwalia A, Alam S, Avey MT, Baker M (2020). The ARRIVE guidelines 2.0: updated guidelines for reporting animal research. PLoS Biol.

[R34] Puxeddu I, Alian A, Piliponsky AM, Ribatti D, Panet A, Levi-Schaffer F (2005). Human peripheral blood eosinophils induce angiogenesis. Int J Biochem Cell Biol.

[R35] Ramon S, Dalli J, Sanger JM, Winkler JW, Aursnes M, Tungen JE (2016). The protectin PCTR1 is produced by human M2 macrophages and enhances resolution of infectious inflammation. Am J Pathol.

[R36] Reichman H, Karo-Atar D, Munitz A (2016). Emerging roles for eosinophils in the tumor microenvironment. Trends Cancer.

[R37] Serhan CN, Levy BD (2018). Resolvins in inflammation: emergence of the pro-resolving superfamily of mediators. J Clin Invest.

[R38] Simard M, Morin S, Ridha Z, Pouliot R (2022). Current knowledge of the implication of lipid mediators in psoriasis. Front Immunol.

[R39] Strid J, Roberts SJ, Filler RB, Lewis JM, Kwong BY, Schpero W (2008). Acute upregulation of an NKG2D ligand promotes rapid reorganization of a local immune compartment with pleiotropic effects on carcinogenesis. Nat Immunol.

[R40] Strid J, Sobolev O, Zafirova B, Polic B, Hayday A (2011). The intraepithelial T cell response to NKG2D-ligands links lymphoid stress surveillance to atopy. Science.

[R41] Urban K, Mehrmal S, Uppal P, Giesey RL, Delost GR (2021). The global burden of skin cancer: a longitudinal analysis from the Global Burden of Disease Study, 1990-2017. JAAD Int.

[R42] Wong DT, Bowen SM, Elovic A, Gallagher GT, Weller PF (1999). Eosinophil ablation and tumor development. Oral Oncol.

[R43] Workman P, Aboagye EO, Balkwill F, Balmain A, Bruder G, Chaplin DJ (2010). Guidelines for the welfare and use of animals in cancer research. Br J Cancer.

[R44] Yasukawa A, Hosoki K, Toda M, Miyake Y, Matsushima Y, Matsumoto T (2013). Eosinophils promote epithelial to mesenchymal transition of bronchial epithelial cells. PLoS One.

[R45] Yeakley JM, Shepard PJ, Goyena DE, VanSteenhouse HC, McComb JD, Seligmann BE (2017). A trichostatin A expression signature identified by TempO-Seq targeted whole transcriptome profiling. PLoS One.

[R46] Yu C, Cantor AB, Yang H, Browne C, Wells RA, Fujiwara Y (2002). Targeted deletion of a high-affinity GATA-binding site in the GATA-1 promoter leads to selective loss of the eosinophil lineage in vivo. J Exp Med.

