# Supplementary figures and images for: Eosinophils and Bioactive Lipid Mediators Regulate Skin Inflammation and Cancer Growth
Source: J Invest Dermatol. Author manuscript; Available in PMC 2026 Jun 15. (PMC7619174; doi:10.1016/j.jid.2025.04.015)

**a**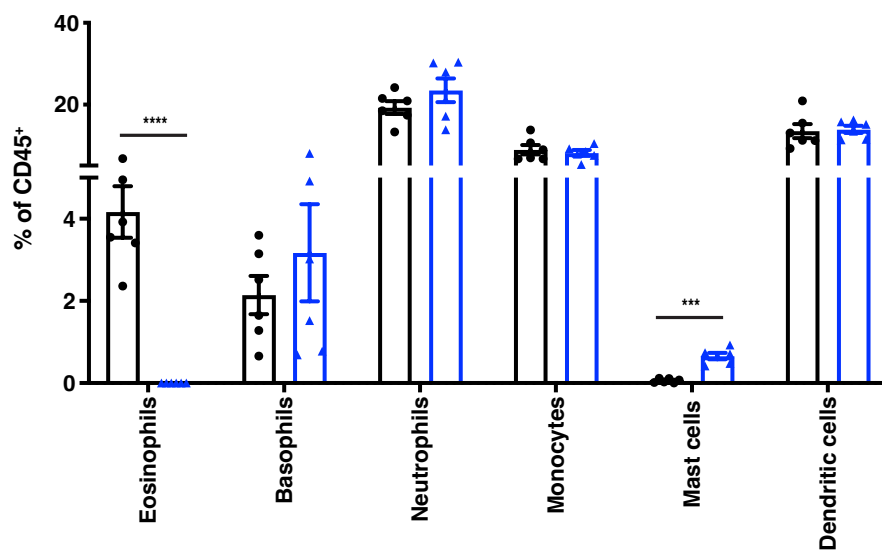**b**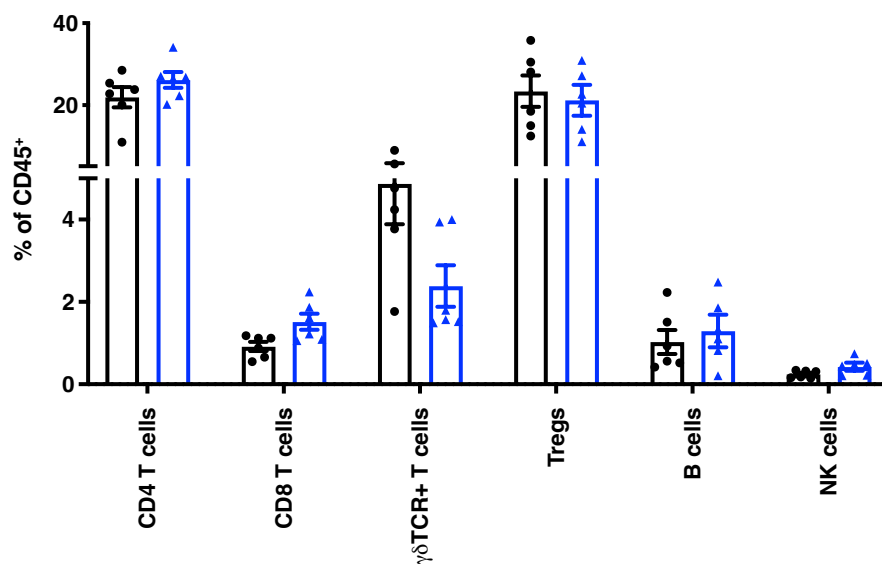

Supplement: Fig. S1 [file EMS214222-supplement-Fig__S1.pdf]

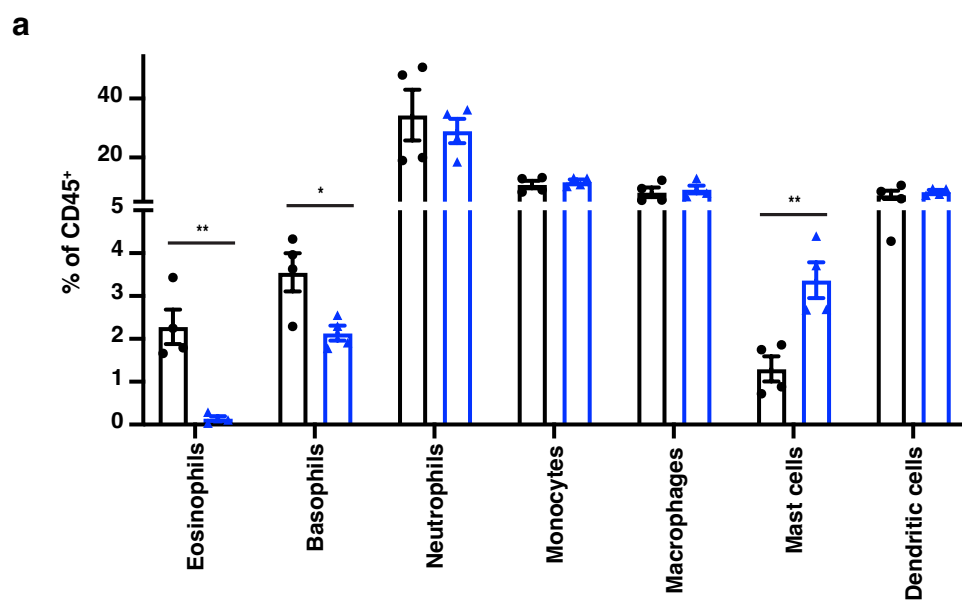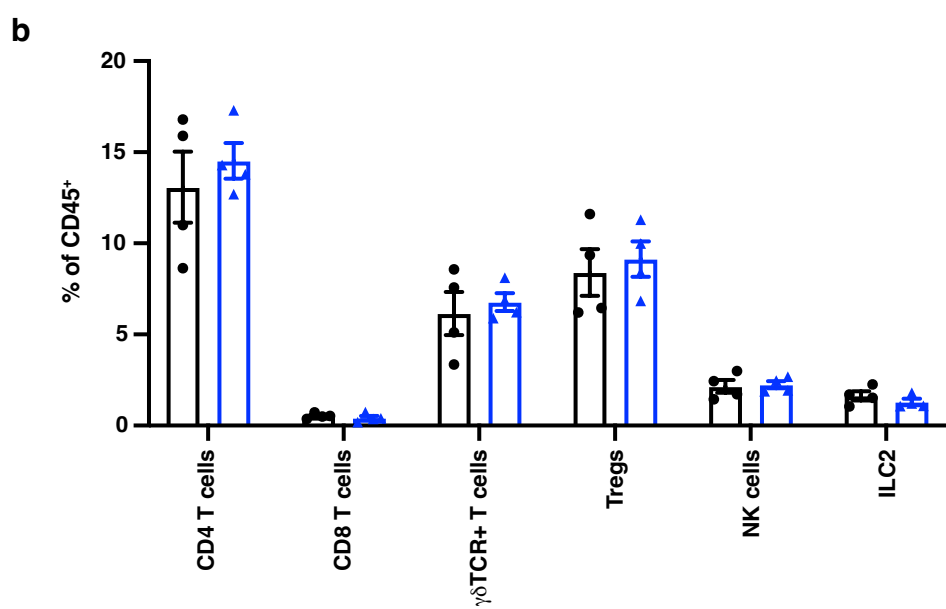

Supplement: Fig. S2 [file EMS214222-supplement-Fig__S2.pdf]

### Unique gene expression in **iSkin** eosinophils

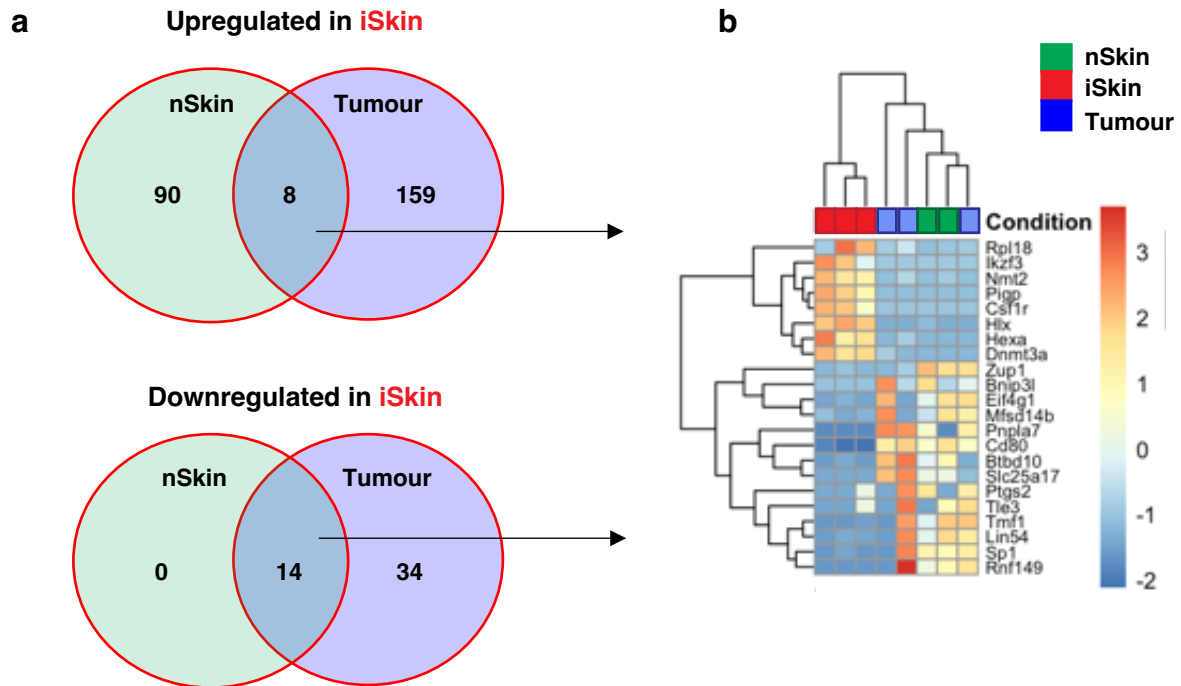

### Unique gene expression in **Tumour** eosinophils

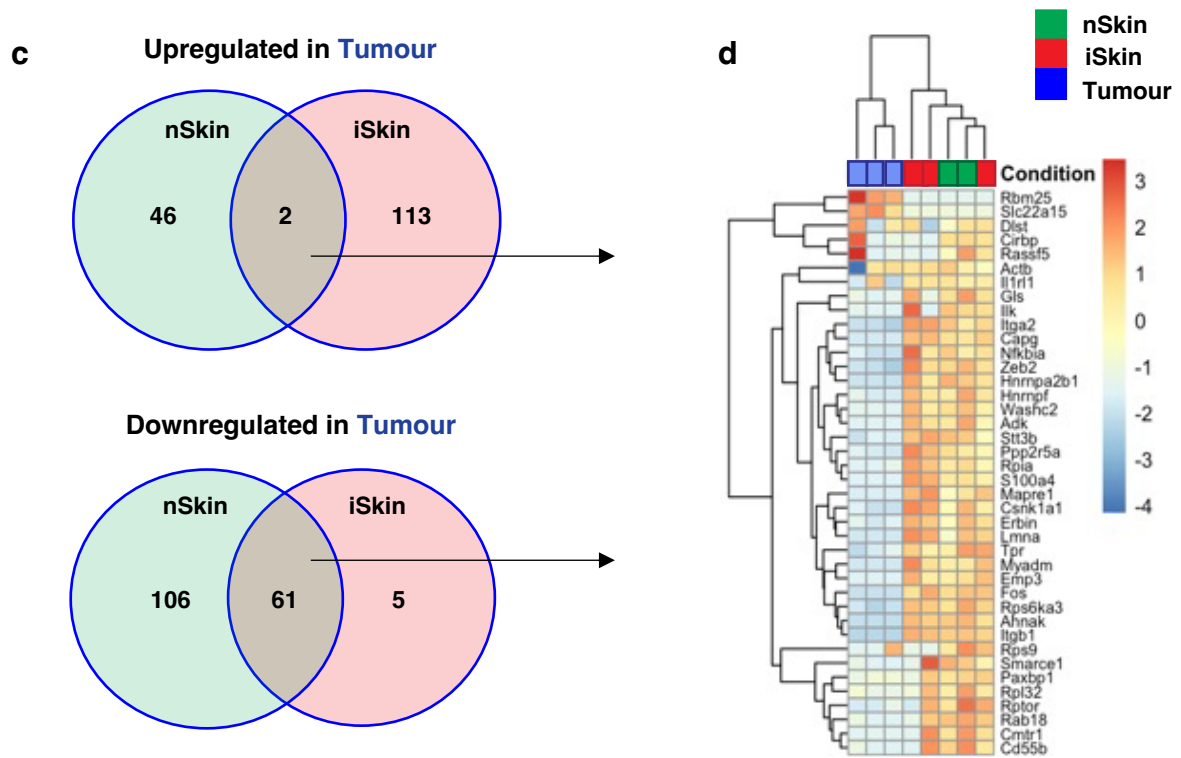

Supplement: Fig. S3 [file EMS214222-supplement-Fig__S3.pdf]

**a**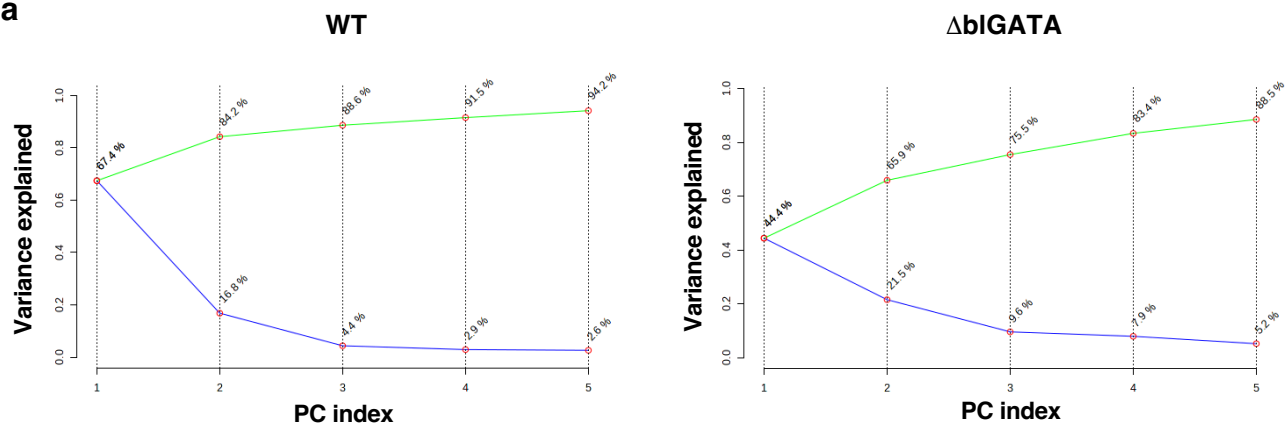**b**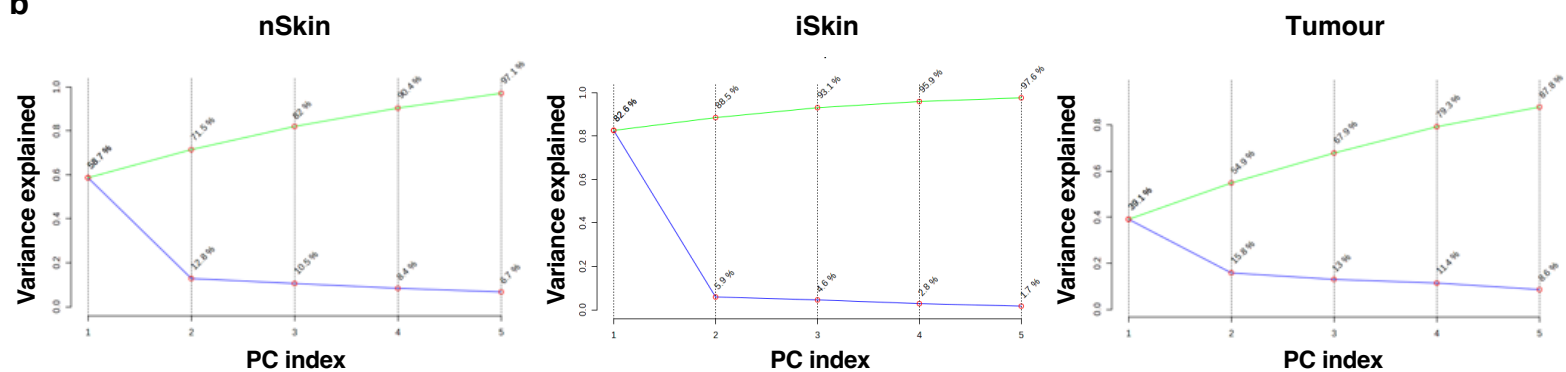**c**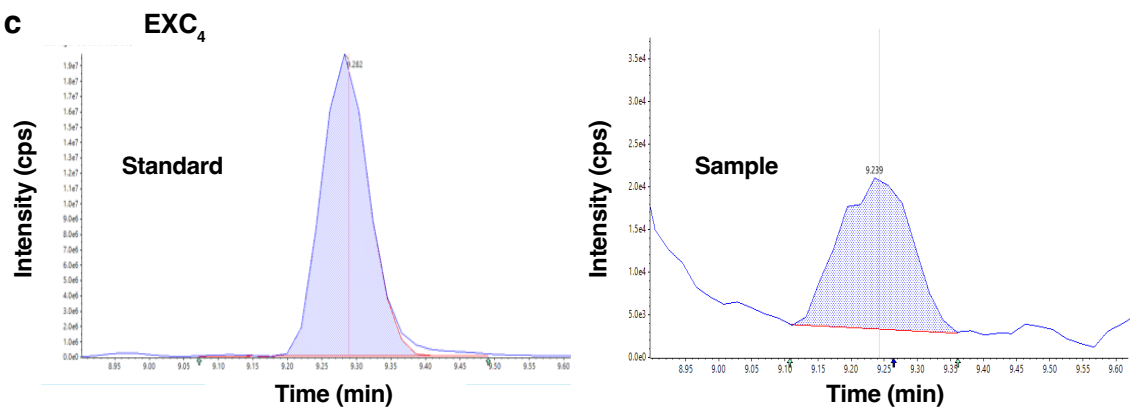**d**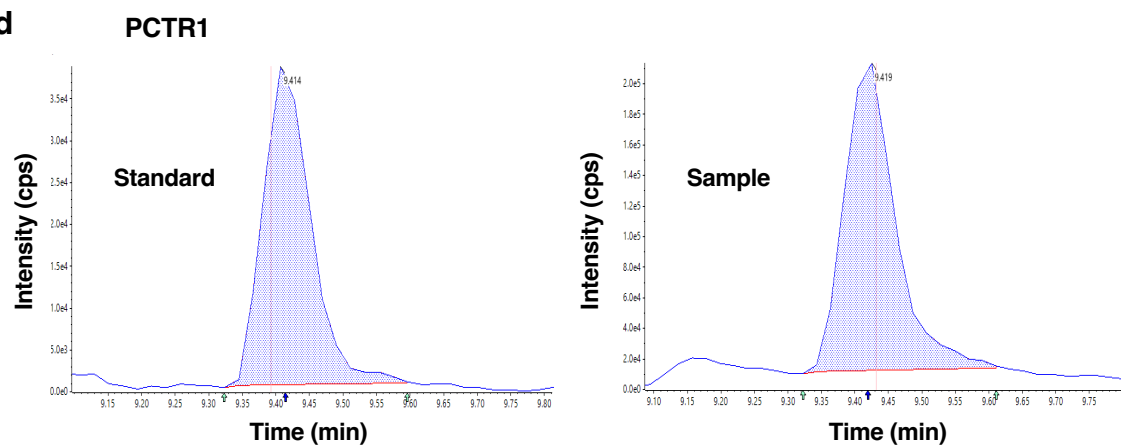

Supplement: Fig. S4 [file EMS214222-supplement-Fig__S4.pdf]

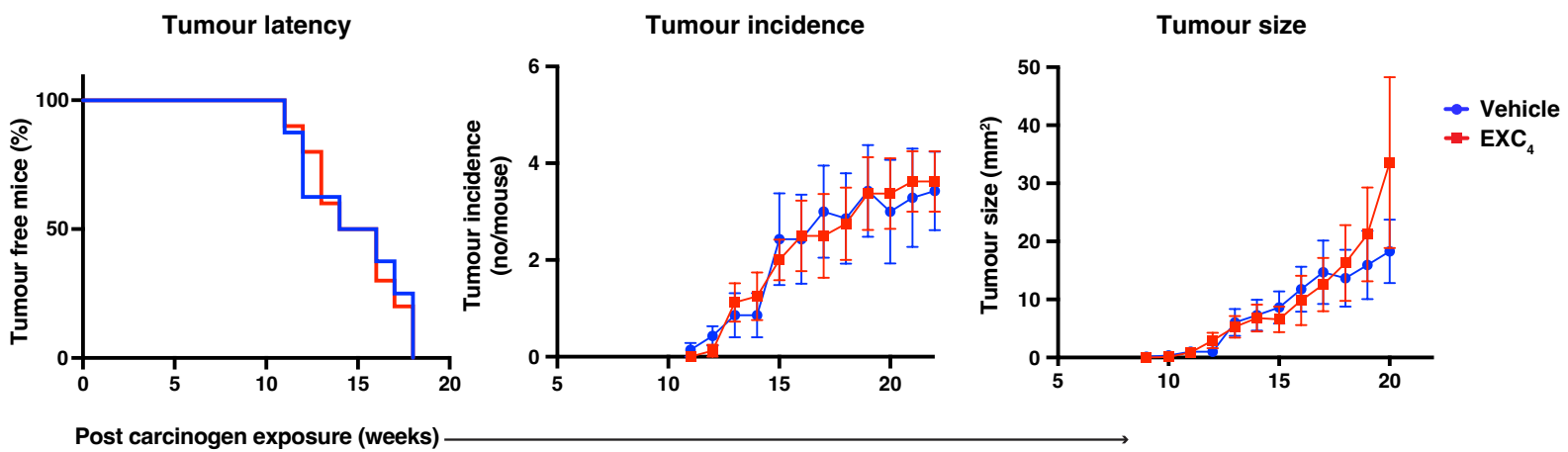

Supplement: Fig. S5 [file EMS214222-supplement-Fig__S5.pdf]
