# Supplementary material for: Eosinophils and Bioactive Lipid Mediators Regulate Skin Inflammation and Cancer Growth
Source: J Invest Dermatol. Author manuscript; Available in PMC 2026 Jun 15. (PMC7619174; doi:10.1016/j.jid.2025.04.015)
Supplement: Supplementary text [file EMS214222-supplement-Supplementary_text.docx]

**Supplementary material**

**Materials and methods**

**Lipid mediator quantification**

Snap frozen skin tissues were placed in 1mL of Methanol with deuterated internal standards (IS) (d_4_-LTB_4_, d_5_-MaR1, d_5_-MaR2, d_4_-PGE_2_, d_5_-LXA_4_, d_5_-RvD_3_, d_5_-RvD2, d_4_-RvE1, d_5_-17R-RvD1, d_5_-LTE_4_, d_5_-LTD_4_ and d_5_-LTC_4_), representing the chromatographic regions of interest, were added to facilitate lipid mediator identification and quantification. Tissues were homogenized using a glass dounce and placed at -20 ^o^C for at least 45 minutes. These were centrifuged and supernatants were extracted, and lipid mediators were quantified using the protocol described in (Dooley et al., 2024) with minor modifications. Briefly, supernatants were subjected to solid-phase extraction using the ExtraHera system (Biotage) and Isolute C18 500mg columns (Biogate). Methyl formate and methanol fractions were collected, brought to dryness, and suspended in phase (methanol/water, 1:1. vol/vol) for injection on a Shimadzu LC-20AD HPLC and a Shimadzu SIL-20AC autoinjector, paired with a QTrap 6500+ (Sciex). Analysis of mediators isolated in the methyl formate fraction was conducted as follows: an Agilent Poroshell 120 EC-C18 column (100 mm × 4.6 mm × 2.7 µm) was kept at 50 °C and mediators eluted using a mobile phase consisting of methanol/water/acetic acid of 20:80:0.01 (vol/vol/vol) that was ramped to 50:50:0.01 (vol/vol/vol) over 0.5 min and then to 80:20:0.01 (vol/vol/vol) from 2 min to 11 min, maintained till 14.5 min and then rapidly ramped to 98:2:0.01 (vol/vol/vol) for the next 0.1 min. This was subsequently maintained at 98:2:0.01 (vol/vol/vol) for 5.4 min, and the flow rate was maintained at 0.5 ml/min. In the analysis of mediators isolated in the methanol fraction, the initial mobile phase was methanol/water/acetic acid of 20:80:0.5 (vol/vol/vol) which was ramped to 55:45:0.5 (vol/vol/vol) over 0.2 min and then to 70:30:0.5 (vol/vol/vol) over 5 min and then ramped to 80:20:0.5 (vol/vol/vol) for the next 2 min. The mobile phase was maintained for 3 min and ramped to 98:2:0.5 (vol/vol/vol) for 2 min. QTrap 6500+ was operated using a multiple reaction monitoring (MRM) method. Each lipid mediator was identified using the following criteria: (1) matching retention time to synthetic or authentic standards (±0.05 min), (2) signal/noise ratio ≥ 5 as previously published (PMID: 38718314). Data was analyzed using validated protocols (Dooley et al., 2024) and Sciex OS v3.0. Chromatograms were reviewed using the AutoPeak algorithm, using ‘low’ smoothing setting and signal to noise ratios were calculated using the relative noise algorithm. External calibration curves were used to quantify identified mediators. Where available calibration curves were obtained for each mediator using synthetic compound mixtures that gave linear calibration curves with R^2^ values of 0.98–0.99. These calibration curves were then used to calculate the abundance of each mediator per 20mg of tissues. Where synthetic standards were not available for the construction of calibration curves, calibration curves for mediators with similar physical properties (e.g. carbon chain length, number of double bonds, number of hydroxyl groups and similar elution time) were used.

**References**

Dooley M, Saliani A, Dalli J. Development and Validation of Methodologies for the Identification of Specialized Pro-Resolving Lipid Mediators and Classic Eicosanoids in Biological Matrices. J Am Soc Mass Spectrom 2024;35(10):2331-43.

**Figure legends**

**Supplementary Figure S1 | Eosinophils do not regulate tumour leukocyte infiltration but subtly affects the relative abundance of tumour immune subpopulations**

WT and ΔdblGATA mice underwent inflammation-driven carcinogenesis (DMBA-TPA) and at week 20 tumour tissue was collected and analysed by flow cytometry (n=6/group). Abundance of infiltrating myeloid (a) lymphoid (b) cell subsets shown as percentage of total CD45^+^ leukocytes. Individual subsets identified as indicated in materials and methods. Data expressed as mean ± SEM. Statistics by unpaired Student’s t-test; ***p<0.001, ****p<0.0001.

**Supplementary Figure S2 | Eosinophils do not regulate overall leukocyte skin infiltration but subtly affects the relative abundance of immune subpopulations during skin inflammation**

WT and ΔdblGATA mice were exposed topically to the inflammatory agent TPA on the dorsal side of the ear skin (2x/week for 2 weeks) and iSkin collected 48h after last TPA treatment and analysed by flow cytometry (n=4/group). Abundance of skin infiltrating myeloid (a) lymphoid (b) cell subsets shown as percentage of total CD45^+^ leukocytes. Individual subsets identified as indicated in materials and methods. Data expressed as mean ± SEM. Statistics by unpaired Student’s t-test; *p<0.05, **p<0.01.

**Supplementary Figure S3 | Unique gene expression of eosinophils in inflamed skin and tumour**

Eosinophils were FACS sorted as CD45^+^Siglec-F^+^CD11b^+^ live leukocytes from nSkin (n=2), iSkin (n=3) and tumour (n=3) of WT mice and RNA isolated, processed and sequenced using TempO-Seq. nSkin samples were collected from naïve resting mice, iSkin following topical exposure to TPA (2x/week for 2 weeks) and tumour tissue following DMBA-TPA carcinogenesis. (a) Venn diagrams show upregulated and downregulated differentially expressed genes uniquely in iSkin eosinophils compared with nSkin and tumour eosinophils. (b) Heatmaps illustrate the genes upregulated or downregulated in iSkin eosinophils compared with both nSkin and tumour (the overlapping genes in the Venn diagrams in (a)). (c) Venn diagrams show upregulated and downregulated differentially expressed genes uniquely in tumour eosinophils compared with nSkin and iSkin eosinophils. (d) Heatmaps illustrate the genes upregulated or downregulated in tumour eosinophils compared with both nSkin and iSkin (the overlapping genes in the Venn diagrams in (c)). Each heatmap row represents a single gene and each column represents one sample. Red indicates high relative expression, and blue indicates low relative expression.

**Supplementary Figure S4 | Analysis of bioactive lipid mediators by LC-MS/MS**

The bioactive lipid mediator profiles of nSkin (n=4), iSkin (n=4) (induced by topical TPA) and cSCC tumour tissue (n=5) (induced by DMBA-TPA carcinogenesis) were analysed by LC-MS/MS from both WT and ΔdblGATA mice. Scree plots show the variance explained by the principle components in the PCA analysis (shown in Figure 5a,b) of (a) nSkin, iSkin and cSCC in WT and ΔdblGATA mice and (b) for the individual tissue conditions for WT and ΔdblGATA mice. The green line shows the accumulated variance explained and the blue line the variance explained by individual principal components. (c,d) Representative chromatograms for EXC_4_ and PCTR1, left panel for each mediator are chromatograms from synthetic standards with right chromatograms obtained from iSkin samples.

**Supplementary Figure S5 | Topical EXC_4_ has no effect on tumour susceptibility**

ΔdblGATA mice (n=8/group) underwent DMBA-TPA inflammation-driven carcinogenesis with/without additional topical EXC_4_ treatment twice a week. Tumour susceptibility is expressed as tumour latency (time to appearance of first tumour), tumour incidence (average number of tumours per mouse) and tumour area (average tumour size per mouse). Data are expressed as mean ± SEM and statistical significance tested by Log-rank (Mantel-Cox) test for tumour latency and linear regression for tumour incidence and area. There was no significant difference between groups.
